# Molecular exploration of hidden diversity in the Indo-West Pacific sciaenid clade

**DOI:** 10.1371/journal.pone.0176623

**Published:** 2017-04-28

**Authors:** Pei-Chun Lo, Shu-Hui Liu, Siti Azizah Mohd Nor, Wei-Jen Chen

**Affiliations:** 1 Institute of Oceanography, National Taiwan University, Taipei, Taiwan; 2 School of Biological Sciences, Universiti Sains Malaysia, Penang, Malaysia; Southwest University, CHINA

## Abstract

The family Sciaenidae, known as croakers or drums, is one of the largest perciform fish families. A recent multi-gene based study investigating the phylogeny and biogeography of global sciaenids revealed that the origin and early diversification of this family occurred in tropical America during the Late Oligocene—Early Miocene before undergoing range expansions to other seas including the Indo-West Pacific, where high species richness is observed. Despite this clarification of the overall evolutionary history of the family, knowledge of the taxonomy and phylogeny of sciaenid genera endemic to the Indo-West Pacific is still limited due to lack of a thorough survey of all taxa. In this study, we used DNA-based approaches to investigate the evolutionary relationships, to explore the species diversity, and to elucidate the taxonomic status of sciaenid species/genera within the Indo-West Pacific clade. Three datasets were herein built for the above objectives: the combined dataset (248 samples from 45 currently recognized species) from one nuclear gene (*RAG1*) and one mitochondrial gene (*COI*); the dataset with only *RAG1* gene sequences (245 samples from 44 currently recognized species); and the dataset with only *COI* gene sequences (308 samples from 51 currently recognized species). The latter was primarily used for our biodiversity exploration with two different species delimitation methods (Automatic Barcode Gap Discovery, ABGD and Generalized Mixed Yule Coalescent, GMYC). The results were further evaluated with help of four supplementary criteria for species delimitation (genetic similarity, monophyly inferred from individual gene and combined data trees, geographic distribution, and morphology). Our final results confirmed the validity of 32 currently recognized species and identified several potential new species waiting for formal descriptions. We also reexamined the taxonomic status of the genera, *Larimichthys*, *Nibea*, *Protonibea* and *Megalonibea*, and suggested a revision of *Nibea* and proposed a new genus *Pseudolarimichthys*.

## Introduction

Over the past few decades, the development of new and diverse DNA-based approaches has made great advances in systematic biology at all taxonomic levels [1–5]. For example, phylogenetic inference based on evidence from multiple gene loci (known as phylogenomic approach) has been utilized for reconstructing the well-supported “Tree of Life” that provides a comparative framework for tests of hypotheses of organismal evolution and for classification of living organisms [1]. On the other hand, the approach of DNA barcoding that uses DNA sequences from a short fragment of mitochondrial cytochrome oxidase subunit I (*COI*) gene as “barcodes” for bio-identification is also well established [6]. These approaches have increased the pace and rigor of biodiversity assessments, especially for traditional methods (e.g., morphological) which sometimes fail to provide a sound resolution [1, 7]. Other obvious advantages include ease of use, applicability at any stage of development/state of the specimen as well as transmissibility [8].

In this study, we conducted a DNA-based survey on a diverse group of predominantly marine/brackish fishes from the family Sciaenidae, commonly known as croakers or drums [9]. This family contains 68 currently recognized genera and approximately 292 species, distributed in temperate and tropical regions worldwide [10–13]. Most of the species are targeted by local fisheries and their global fishery production in the past two decades is around one million ton per year [14]. However, many sciaenid species are vulnerable to overfishing [15–20] and habitat degradation [18, 21, 22]. In 2007, the IUCN-Species Survival Commission identified this family as a conservation priority and called for the urgent establishment of a Sciaenidae Red List Authority to assess the risk of extinction of all sciaenid species and to recommend actions needed for the next decade to ensure their well-being [23]. However, without a solid framework for the systematics of the Sciaenidae, prospective work with species stock management and conservation cannot be properly understood.

Recently, Lo et al. [24] reconstructed the first comprehensive phylogeny of global sciaenids with rigorous taxonomic and molecular character sampling (6619 bp from two mitochondrial and four nuclear genes for 93 taxa). The constructed phylogeny was subsequently used as a framework to examine their origin and historical biogeography. They found that the Sciaenidae first originated and diversified in the tropical American waters in the Late Oligocene and dispersed outward in the Early Miocene, hypothesizing that the ancestor of the Indo-West Pacific endemic sciaenid genera underwent a single invasion event to the Indo-West Pacific (IWP) that evolved to form a diversified clade that today contains approximately 91 species [10, 24]. However, knowledge of the taxonomy and phylogeny of the sciaenids within this IWP clade is still limited due to lack of a thorough survey of all taxa. Most previous studies have focused on the investigation of the population structure of certain species and DNA barcoding studies at a regional scale [25–28], and in the past decade, only two taxonomic studies have reported new species [29, 30]. Possible cryptic species may exist in the IWP realm that await further discovery.

Traditionally, biodiversity exploration relies on morphology-based species identification. However, such a task is challenging for congeneric species in the Sciaenidae because of similarity in their external morphology and overlapping meristic counts. The use of DNA-based approaches for species identification represents a novel perspective to complement traditional taxonomic practices that are generally time consuming even for experts. In the present study, a combined approach with multi-locus phylogenetic and DNA barcoding analyses was employed to investigate evolutionary relationships, explore species diversity, and to elucidate the taxonomic status of sciaenid species/genera within the IWP clade.

## Materials and methods

### Ethics statement

**Fig 1:**
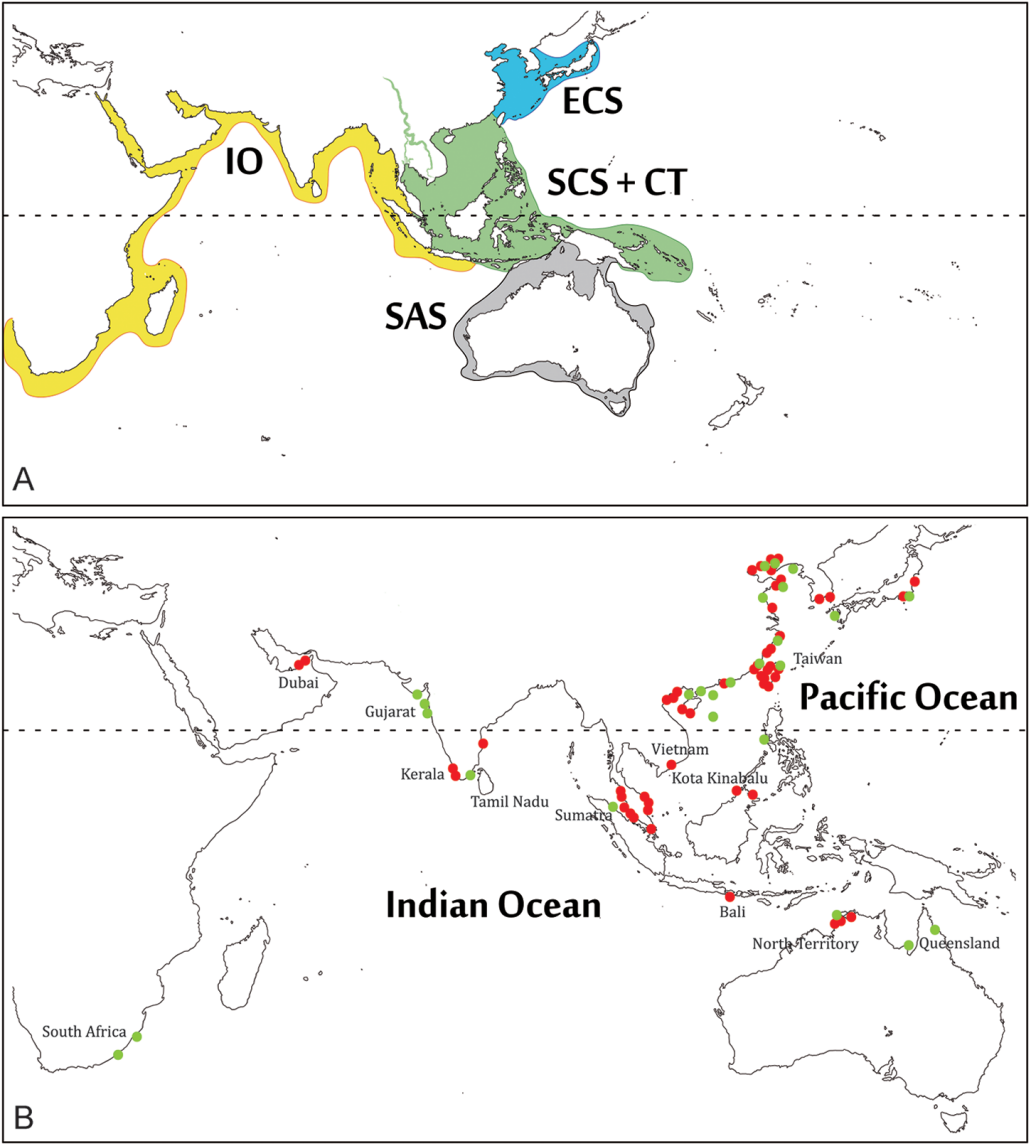
**The four regions used for considering the sympatry of sister OTUs (A), and map of the Indo-West Pacific region indicating the sampling sites for the taxa examined in the present study (B).** Abbreviations: IO, Indian Ocean; ECS, East China Sea; SCS, South China Sea; CT, Coral Triangle region; SAS, Sahul Shelf and Australia. Dots in different color represent the examined samples from different sources. Red dots: our own collections; green dots: online databases.

The research was performed at National Taiwan University in accordance with the National Taiwan University’s guidelines regarding animal research. As this project did not involve experiments on live fishes, no ethics statement is required. Most of the specimens examined in the present study were purchased from local fish markets, fish landing sites or directly from professional fishermen (see Fig 1 and S1 Table for the details); others were museum specimens. The taxonomic sampling was completed through legal tissue donations from International Research Institutes (see “acknowledgments” section).

### Sample collection

The sciaenid samples (n = 249) of 45 recognized species from the IWP clade were collected from 96 localities (Fig 1 and S1 Table). The collected specimens were identified using morphological characters, primarily based on the *Fishes of Taiwan* [31] and FAO (Food and Agriculture Organization of the United Nations) and other species identification guides [32, 33]. After identification, a small piece of muscle-tissue was excised from each specimen, preserved in 95% ethanol, and for most of the samples, voucher specimens (n = 143) were preserved and deposited in the National Taiwan University Museums (NTUM), Taipei, Taiwan and in the Centre for Marine and Coastal studies in the Universiti Sains Malaysia (USM_CEMACS), Penang, Malaysia (S1 Table).

### DNA extraction, PCR amplification and sequencing

Total genomic DNA was extracted from each tissue sample using DNeasy Blood and Tissue Kit (Qiagen, Hilden, Germany) or LabTurbo DNA Mini Kit LGD480-220, (TAIGEN Bioscience Corporation, Taipei, Taiwan) following the manufacturer’s protocols. Polymerase chain reaction (PCR) was used to amplify the following targeted gene markers: mitochondrial cytochrome c oxidase I (*COI*) gene and exon 3 of nuclear recombination-activating gene 1 (*RAG1*). These two gene markers were selected for their ability to provide valuable phylogenetic information (*RAG1*) and to delimit species (*COI*) based on previous studies investigating fish systematics [24, 34–38]. These two markers were also part of the gene set used in a previous study on the Sciaenidae [24] and provide consistent results from PCR to sequencing processes. Information on the primers for these gene markers are listed in S2 Table. Laboratory protocols to obtain the sequences followed Lo et al. [24]. The short amplicons from *COI* were sequenced with only forward or reverse primer because current Sanger sequencing technique has no issue of sequencing accuracy for a relatively short fragment. However, the longer amplicons from *RAG1* were sequenced with both forward and reverse primers in order to robustly assemble the sequences.

The obtained DNA sequences were edited with the sequence assembly and alignment software, CodonCode Aligner v.6.0.2 (CodonCode Corporation, Dedham, MA, USA) and Se-Al v.2.0 [39]. The sequences (usually at both extreme ends) with low quality or problematic base calls, i.e. below Q (phred quality value) 20, were verified visually and trimmed. In addition, the possibility of sequencing errors resulting from sample mix-up or contamination was checked by comparing the topologies of the resulting phylogenetic trees individually inferred from each gene fragment and/or comparing to GenBank archived sequences of a putatively closely related taxon using BLAST (http://www.ncbi.nlm.nih.gov/BLAST/). Manipulation and/or species identification errors were further checked by examining the voucher specimens if possible or the additional sequence of a second exemplar from the same (or nearby) locality of the sample collection. The newly obtained sequences were deposited in the NCBI GenBank under accession numbers, KX777879 –KX778099 (221 *COI* sequences) and KX777663 –KX777878 (216 *RAG1* sequences).

### Analytical methods

#### Molecular datasets and phylogenetic reconstruction

For the molecular analyses, three different datasets were compiled and used: 1) *COI* dataset (308 samples) comprising the *COI* gene sequences newly obtained in the present study (n = 219) and those retrieved from three online databases (see section below) (n = 89); 2) *RAG1* dataset (245 samples) including the *RAG1* gene sequences obtained in the present study (n = 214) plus several from GenBank (n = 31); 3) the combined dataset (248 samples) including the sequences from the common taxon set of the first and second datasets (n = 245) plus the *COI* sequences from three taxa (*Collichthys niveatus* CN545, *Johnius heterolepis* CN557 and *Nibea soldado* WJC1935) whose *RAG1* sequences were missing due to the PCR/sequencing failure of the gene. The samples from these three compiled datasets represented taxa from 18 of 25 IWP endemic sciaenid genera. Six non-IWP, but closely related sciaenids, *Aplodinotus grunniens*, *Pseudotolithus brachygnathus*, *P*. *elongatus*, *P*. *senegalensis*, *Pteroscion peli* and *Totoaba macdonaldi* were selected as outgroups following Lo et al. [24] for the phylogenetic analyses. The distant outgroup, *Aplodinotus grunniens*, was used to root the inferred phylogenetic trees.

The sequence alignment for each gene dataset was achieved with the automatic multiple alignment program MUSCLE [40], then adjusted manually by eye using the software Se–Al v2.0 [39]. The descriptive statistics (sequence variation, base composition, genetic distances, etc.) for the compared sequences of each gene were calculated using the tools in PAUP*4.0a147 [41]. To infer phylogenetic trees, the partitioned Maximum Likelihood (ML) method [42], as implemented in the sequential and parallel program RAxML v.0.93 [43] was used. Because RAxML only provides GTR-related models of rate heterogeneity for nucleotide data [43], the nucleotide substitution model GTR+*Γ*+I [44] was employed for the analyses by following the decision from our previous study [24]. For each ML search, five independent runs were conducted and the final tree with the best ML score was selected among the five ML trees of these runs. Nodal support was assessed with bootstrapping [45] under the ML criterion, based on 1000 pseudo-replicates generated from each of the five separate runs. All RAxML analyses with bootstrapping were conducted with the CIPRES Science Gateway at http://www.phylo.org [46].

#### Biodiversity assessment through species delimitation analyses

To explore the sciaenid species diversity within the IWP clade, two DNA-based analytical methods applying different algorithms of species delimitation were primarily used (see below). Both methods determine the sequence clusters, as operational taxonomic units (OTUs) or putative species without *a priori* species hypothesis based on the *COI* sequence dataset compiled in this study. In this dataset, 219 new plus 89 published sequences retrieved from NCBI GenBank [47], BOLD system (The Barcode of Life Data System; [48]) and CRYOBANK (Taiwan Wildlife Genetic Material Cryobank; [49]) were included (see list of examined taxa in S1 Table).

The Automatic Barcode Gap Discovery (ABGD) [50] method is an exploratory tool based on pairwise genetic distances. Under the premise that the intraspecific diversity of the *COI* gene is lower than the interspecific diversity, this tool applies clustering algorithms to automatically detect the significance between intra- and inter-specific variations (called barcode gap). A default setting of relative gap width (X) as 1.5, intraspecific divergence (P) value from 0.001 to 0.1 with 50 steps under both available distance metrics, JC69 [51] and the Kimura-2-parameter distance model (K2P) [52], were employed at the ABGD web interface (http://wwwabi.snv.jussieu.fr/public/abgd).

The Generalized Mixed Yule Coalescent (GMYC) [53] method is a likelihood solution that calculates the transition point between the speciation and coalescence processes with the assumption of a constant species rate and no extinction on an ultrametric gene tree. A single-threshold GMYC analysis was performed using RStudio v.0.99.486 software [54], an integrated development environment for R software v.3.2.1 [55] using the APE [56] and SPLITS [57] packages. An input tree for GMYC method with ultrametric, bifurcating and no zero branch lengths was generated using BEAST v.1.8.3 [58] based on a relaxed molecular clock using a Bayesian approach. Nine independent runs of 5 x 10^7^ generations each were performed. Estimation of trees and divergence time were sampled once every 5000 generations and the parameters of each run were checked for convergence using Tracer v.1.6 [5]. For each run, two inferred ages, 12.8 Ma and 16.6 Ma, respectively according to the previous study [24], were set as calibration points and were used to constrain the tMRCAs of West African sciaenids and endemic IWP sciaenids based on a GTR+*Γ* substitution model to infer the time tree. The burn-in parts of each run (= 10%, i.e., 10^3^ trees per run) were removed and the tree samples from the remaining nine runs were used to reconstruct the maximum clade credibility tree with mean divergence times using TreeAnnotator v.1.8.3 [5].

Congruent results between these two methods are considered as robust support of any OTU as suggested by Carstens et al. [59]. Other species delimitation criteria [60] were taken into account to minimize the bias from these two analyses for the final validation. In this study, we employed four criteria to aid a final decision on the status of any incongruent OTU: reciprocal monophyly found in gene trees (both *COI* and *RAG1* tree), genetic similarity, geographical distributions, and morphology.

Here, monophyly of each incongruent OTU was further evaluated based on the phylogenetic trees reconstructed based on *COI*, *RAG1*, and combined datasets (see method described below). The analysis of unlinked markers (mitochondrial vs. nuclear genes) allowed evaluation of the absence of gene flow between putative species or their presence of reproductive isolation “*in situ*”.

Genetic similarity was evaluated by the K2P genetic distances [52] estimated using PAUP*4.0a147 [41]. The maximum genetic distances within and among each OTU consistently defined from the ABGD analysis were calculated and set as our threshold of evaluation. Multiple OTUs in the same recognized species with less than the maximal sequence divergences would be merged if there were no other evidences against the prior species definition by morphology.

In addition, as the establishment of reproductive isolation between putative species in the field can only be tested in sympatric species [61], we took the sympatry of the sister OTUs into consideration and merged the allopatric sister OTUs as a single one. Because sciaenids are non-coral reef fishes and always have restricted migratory habits; only their pelagic larval stage has potential high dispersal along coastal areas [25, 26, 28, 62]. Thus, we defined four geographic regions based on the landmass or boundaries of coral reefs, currents and levels of regional endemicity, i.e. East China Sea, ECS; South China Sea + Coral Triangle, SCS + CT; Sahul Shelf and Australia, SAS; Indian Ocean, IO (Fig 1). The designation of the sympatry of inferred sister OTUs is based on these four regions.

Finally, morphological measurements and counts of the major diagnostic characteristics of the sciaenid species were obtained following methodologies of Hubbs and Lagler [63]. The compared specimens were from our collected samples (S1 Table) and those from the holotype specimen of *Nibea soldado* (Lacepède 1802) (MNHN 5550). Gill raker counts were taken from the first right-hand side arch of the specimens except where this arch was damaged; denticulate flat plates above and below the rakers were not counted, and the middle raker counts were included in the lower raker numbers.

## Results and discussion

### Characteristics of sequence data

The *COI* dataset comprised 308 sequences retrieved from public databases and those generated from our collected samples (S1 Table). Indels and stop codons were absent from all amplified sequences. Within the *COI* dataset, the total 618 bp contained 311 (50.3%) variable sites, 287 of them parsimony-informative. Most of the variable sites were found in the third codon positions (206/311; 66.2%). All of the sites in the third codon positions were variable and parsimony-informative (S3 Table).

The *RAG1* dataset comprised 245 sequences retrieved from public databases and those generated from our collected samples (S1 Table). Indels and stop codons were absent from all amplified sequences. Within the *RAG1* dataset, the total of 1472 bp contained 348 (23.6%) variable sites, 269 of them parsimony-informative. Most of the variable sites were found in the third codon positions (249/348; 71.6%). About a half of sites in the third codon positions were variable (249/490; 50.8%) and 195 of them were parsimony-informative (S3 Table).

The combined dataset (2090 bp) in the present study contained the sequences of *COI* gene (618 bp) and *RAG1* gene (1472 bp) from a total of 248 examined samples. Indels and stop codons were not found along the sequences. Among the nucleotide sites, 640 were variable (30.6%) and 548 of them were parsimony-informative (S4 Table).

### Inferred phylogenetic trees

**Fig 2:**
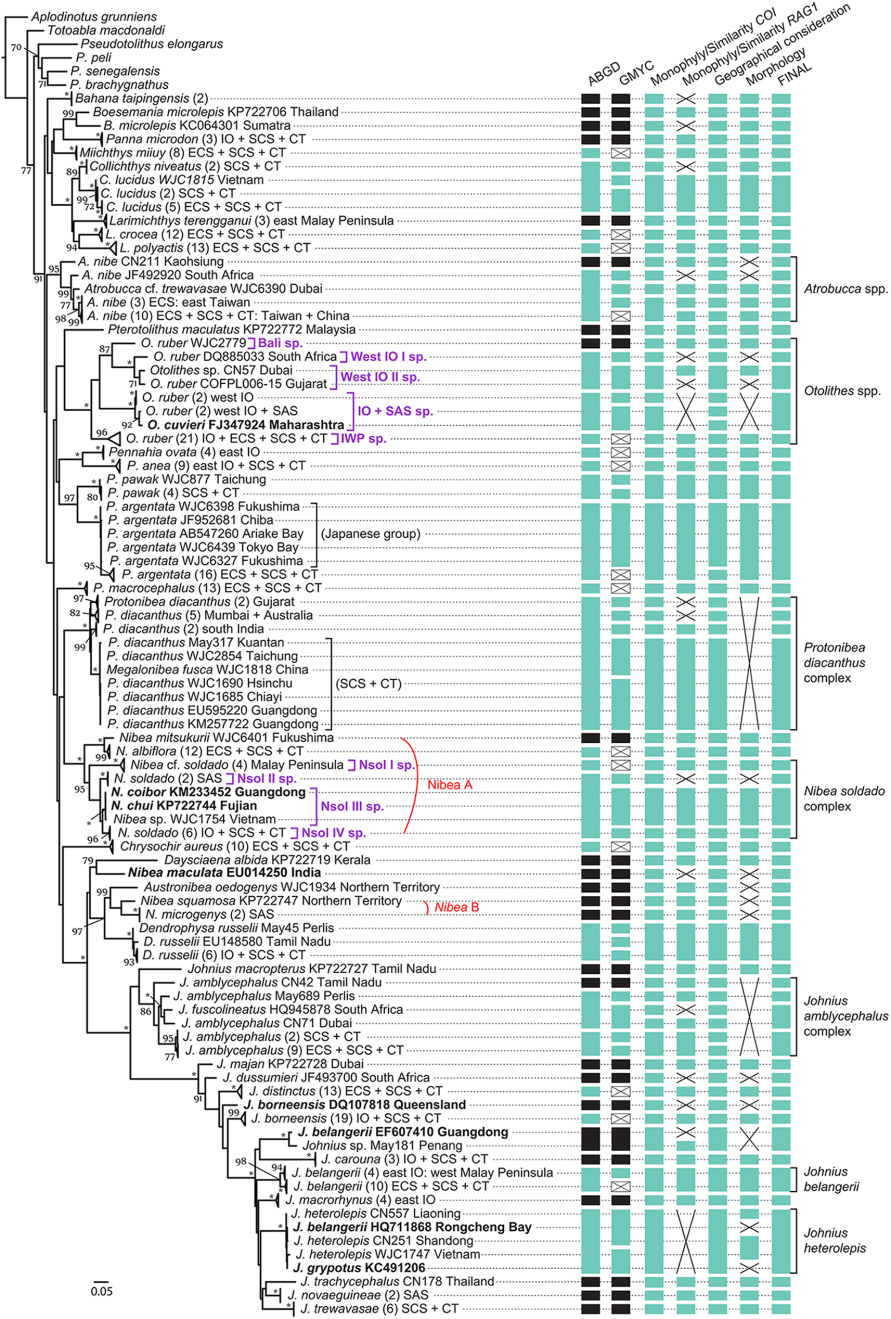
Summarized phylogenetic tree of the IWP sciaenids inferred by partitioned maximum-likelihood method with GTR+*Γ*+*I* nucleotide substitution model based on the *COI* gene dataset and results from species delimitation analyses and other considerations by four additional criteria (see materials and methods). Branch lengths are proportional to the inferred nucleotide substitutions. Numbers at nodes represent bootstrap values in percentage. Values below 70% are not shown; * indicates 100%. Taxa name in bold indicates problematic or probable misidentification samples. Numbers within the parentheses shown after the taxon names indicate number of sequences being contained within each collapsed clade or lineage. The tree is rooted with *Aplodinotus grunniens* according to Lo et al. [24]. Summary of the determined OTUs (vertical bars) is presented on the right side of the phylogenetic tree. Black bar indicates congruent OTUs as suggested by both species delimitation methods (i.e. robust results). White bar with a cross inside indicates the presence of multiple OTUs determined by GMYC in the particular genetic lineage that is incongruent to ABGD result. Missing data are marked by black crosses. Abbreviations for geographic distribution of the taxa: IO, Indian Ocean; ECS, East China Sea; SCS, South China Sea; CT, Coral Triangle region; SAS, Sahul Shelf and Australia.

We tested the monophyly of the IWP sciaenid clade and examined the validity of 18 of 25 IWP endemic sciaenid genera, and 51 of 91 recognized species from the IWP clade. The inferred mitochondrial *COI* gene tree is shown in S1 Fig and summarized in Fig 2. In this tree, the IWP sciaenid clade revealed by Lo et al. [24] is confirmed with high bootstrap support (BS = 91%). While 23 of 31 examined species that contain more than two representative taxa sampled for each species are monophyletic with high bootstrap supports, six others are paraphyletic (*Atrobucca nibe*, *Otolithes ruber*, *Protonibea diacanthus*, *Nibea soldado*, *Johnius amblycephalus*, *J*. *heterolepis*) and two are polyphyletic (*J*. *borneensis* and *J*. *belangerii*) (Fig 2 and S1 Fig).

The nuclear *RAG1* gene analysis involved fewer taxa (245 samples from 44 species) than the *COI* gene analysis. The result showed that the resolution of the relationships among closely related species and within species or species complexes inferred from *RAG1* is less pronounced than that inferred from *COI* because of its conservative rate of gene evolution. However, the *RAG1* gene tree (S2 Fig) still confirms most of the species monophyly found in the *COI* gene tree (S1 Fig) and the results of species delimitations based on *COI* dataset when the concerned sequence data are available (see further discussion below).

**Fig 3:**
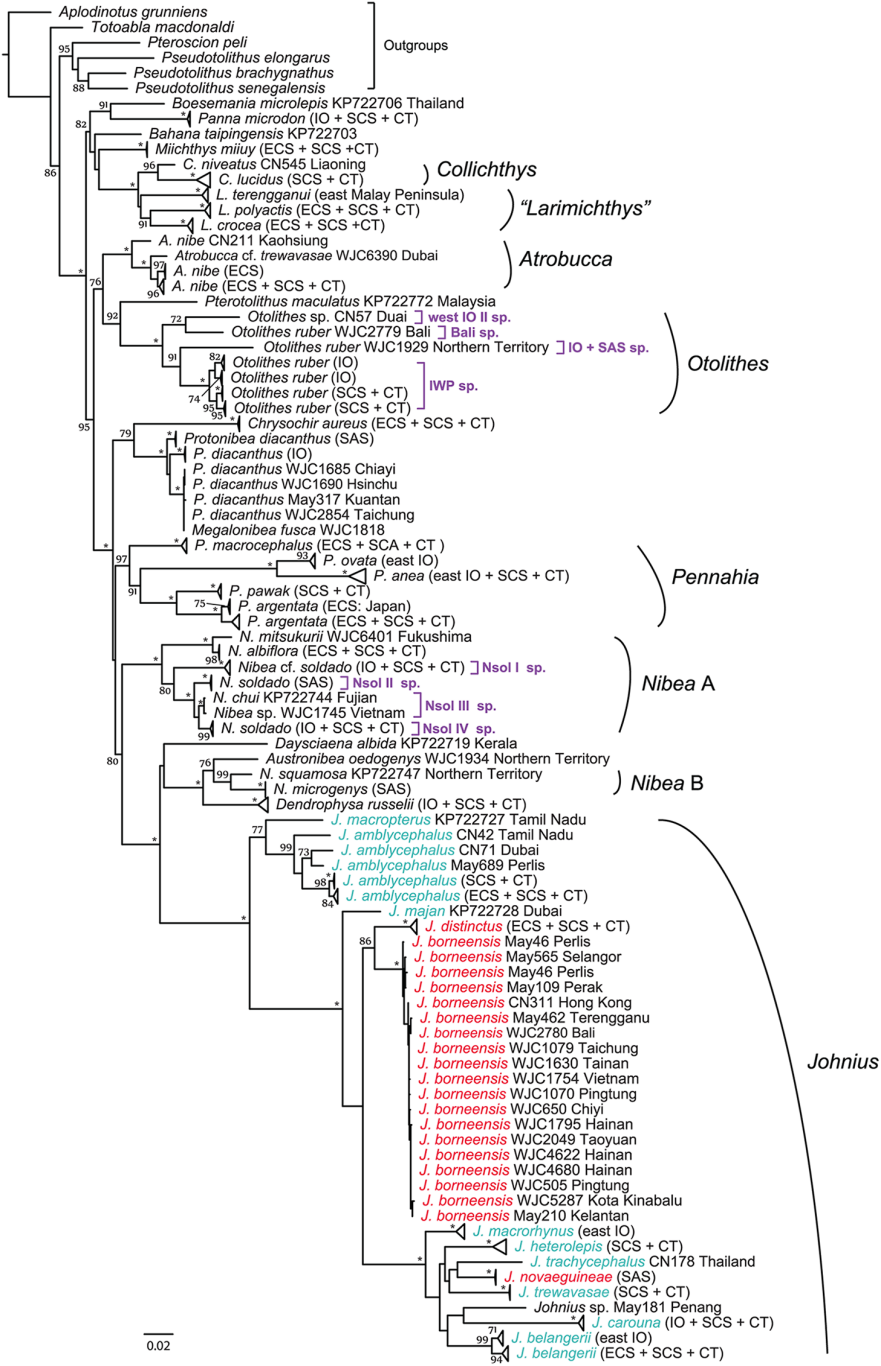
Phylogenetic tree of the IWP sciaenids inferred by partitioned maximum-likelihood method with GTR+*Γ*+*I* nucleotide substitution model based on the combined dataset. Branch lengths are proportional to the inferred nucleotide substitutions. Numbers at nodes represent bootstrap values in percentage. Values below 70% are not shown; * indicates 100%. Different colors in genus *Johnius* show that the taxa are either from the pre-recognized subgenus, *Johnius* (in blue) or subgenus *Johnieops* (in red). The tree is rooted with *Aplodinotus grunniens* according the result shown in Lo et al. [24]. Abbreviations for geographic distribution of the taxa: IO, Indian Ocean; ECS, East China Sea; SCS, South China Sea; CT, Coral Triangle region; SAS, Sahul Shelf and Australia.

The combined gene analysis (Fig 3) contained 248 samples from 44 recognized species including 6 outgroups. The IWP sciaenid clade received the highest bootstrap value (BS = 100%) in the inferred tree. In contrast to the *COI* and *RAG1* individual gene datasets, the combined gene dataset provided better overall signals for resolving both shallow and deep nodes in the inferred phylogenetic tree, as revealed by the presence of a high proportion of well-supported nodes (BP > 80) from the analysis (Fig 3). Here, we primarily use the results from the combined gene tree to evaluate the higher-level taxonomic status of the sciaenids within the IWP clade as discussed below.

### Higher-level taxonomic implications and revised classification

The combined gene tree confirms the monophyly of the following genera where more than two samples and/or recognized species for each of the following genera were included and examined: *Collichthys*, *Atrobucca*, *Otolithes*, *Pennahia*, and *Johnius* (BS = 96–100%; Fig 3).

For the *Larimichthys* (yellow croakers), three out of four recognized species of the genus, including the recently discovered species, *L*. *terengganui*, that is endemic to eastern Peninsula Malaysia [30], were analyzed. While *L*. *polyactis* and *L*. *crocea* appear to be sister-group with strong bootstrap support for the relationship (BS = 91%), the monophyly of the *Larimichthys* when *L*. *terengganui* is included is not supported (BS < 70%; Fig 3). This makes the assignment of the recently described species to the genus *Larimichthys* problematic. As the swimbladder morphology is often considered a key feature for the diagnosis at generic level in sciaenids [64], we further compared the swimbladder morphology of *Larimichthys* spp. as well as their relatives, *Collichthys* spp. (Fig 3). We observed that the swimbladder appendages from these taxa showed significant differences in their pattern and number. The swimbladder appendages of the *L*. *terengganui* are similar to *L*. *pamoides* (not sampled in this study), with two equally short branches without elongation at the ventral limb and each have 26 appendage pairs in total [30]. In contrast, the branches of *Collichthys* spp. and two other *Larimichthys* species, *L*. *polyactis* and *L*. *crocea*, have elongated ventral limbs (at least for one of them) and the number of their appendage pairs is either < 21 (in *Collichthys*) or > 30 (in *L*. *polyactis* and *L*. *crocea*), which do not overlap with *L*. *terengganui* and *L*. *pamoides* [30–32]. Based on the phylogenetic results (i.e. three genetically distinct [or separately evolving] lineages appearing in the *Larimichthys/Collichthys* clade), concomitant with the morphological evidence, we suggest that *L*. *terengganui* and perhaps *L*. *pamoides* should be assigned to a different genus.

For *Johnius* species, Trewavas [65] recognized two subgenera, *Johnius* and *Johnieops*, according to the morphology of their teeth. The lower jaw teeth of *Johnius* spp. are either uniform in size or molariform, while *Johnieops* spp. possess enlarged conical teeth appearing on the inner row of their lower jaw [35, 62]. This morphological difference led Lal Mohan et al. [64] to suggest both subgenera were worthy of generic ranking. Based on our phylogenetic results, however, such classification should be rejected as grouping based on teeth morphology is not supported (Fig 3).

For the “*Nibea”* species, eleven currently recognized species are included in this genus, of which seven were sampled for the analyses in this study. Based on a multi-gene dataset, Lo et al. [24] demonstrated that this genus was polyphyletic and the examined *Nibea* taxa formed two distantly related clades: *Nibea* A and *Nibea* B. *Nibea* A contains the *Nibea* species commonly found in the Northwest Pacific, the South China Sea, and the eastern Indian Ocean, while *Nibea* B contains *Nibea* species restricted to the coasts of New Guinea and northern Australia. The most closely related sciaenid genus to the *Nibea* B is a monotypic genus, *Austronibea*, endemic to Australia, and another monotypic genus, *Dendrophysa*, is sister to them (Figs 2 and 3). With more included taxa than the previous study [24], notably *N*. *mitsukurii* from Japan and *N*. *chui* from Fujian, China, the present phylogeny reveals the same distinct clades for the *Nibea* species (Figs 2 and 3). In addition, a third clade based on a GenBank sequence from *N*. *maculata* collected from India, is found in the *COI* gene tree (Fig 2 and S1 Fig). This particular taxon appears to be the sister group of *Daysciaena albida*, but does not show a close affinity with *Nibea* A or *Nibea* B (Fig 2 and S1 Fig). As the sequence of this species was retrieved from GenBank (accession no. EU014250), and because no further taxonomic and morphological information about the specimen was given in the paper which reported the sequence [66], we were not able to investigate its taxonomic status.

Based on the inferred phylogeny presented here, we suggest separating the “*Nibea”* species into at least two different groups or genera. The genus *Nibea* should be used only for species of the *Nibea* A clade as it includes the type species of the genus, *N*. *mitsukurii*. We suggest that the two *Nibea* B species, *N*. *squamosa* and *N*. *microgenys* examined here, should be included in the newly redefined *Austronibea*. *Austronibea* contains phylogenetically related, morphologically similar, and geographically co-distributed species, including *A*. *oedogenys* (type species), the two *Nibea* B species, plus possibly another northern Australian distributed “*Nibea*” species, *N*. *leptolepis* (not sampled in this study). Our tree also suggests that *Nibea maculata* might represent another (new) genus, which requires further examination when more data are available. Herein, we propose the following revised classification.

Genus *Pseudolarimichthys* gen. nov. Lo, Liu, Mohd Nor and Chen

Type species: *Pseudolarimichthys terengganui* (Seah, Hanafi, Mazlan and Chao 2015)

Diagnosis: This new name applies to *Larimichthys terengganui*. A former genus name “*Larimichthys”* was assigned to this species by Seah et al. [30] who described it without giving a clear reason. Our phylogenetic results show it is only distantly related to *Larimichthys* species (Figs 2 and 3). This genus can be generally distinguished from other sciaenid genera by having a specific carrot-shaped swimbladder with a number of branched appendages that contain well-developed dorsal and ventral limbs along its entire lateral side, and having cycloid scales on anterior half of body. This genus can be further distinguished from its two related genera (*Larimichthys* Jordan and Starks 1905 and *Collichthys* Günther 1860) by deep genetic differences (Figs 2 and 3) and the pattern of swimbladder morphology which has two equally short branches without elongation of the ventral limb and 26 appendage pairs in total (versus > 30 and < 21 in *Larimichthys* and *Collichthys*, respectively).

Etymology: *Pseudolarimichthys*. A noun in apposition. Latin *pseudo*, meaning false, and similar to species of *Larimichthys* of the originally described species [30].

Composition: The genus contains *P*. *terengganui* and possibly “*Larimichthys” pamoides* (Munro 1964). The inclusion of the latter species in the genus needs to be further studied.

Distribution: East coast of Peninsular Malaysia.

Genus *Austronibea* Trewavas 1977, new definition

Type species: *Austronibea oedogenys* Trewavas 1977.

Diagnosis: The revised genus also includes species of *Nibea* clade B according to our molecular phylogenetic results (Figs 2 and 3) and in Lo et al. [24]. The diagnostic characters used for the type species can, in part, be considered for characterizing this genus. This species and “*Nibea*” species share most of the diagnostic characters including meristic counts that can be used to distinguish them from all other sciaenid species [32] (see below). However, the species of the redefined genus have a distinctly inferior mouth, which is different from the species of *Nibea* clade A (mouth terminal or slightly inferior). This genus can be diagnosed from the related genus, *Dendrophysa* Trewavas 1964, by the presence of a mental barbel in the latter.

Composition: The redefined genus includes the type species *Austronibea oedogenys*, as well as *A*. *squamosa* (Sasaki 1992) and *A*. *microgenys* (Sasaki 1992) (removed from *Nibea*, as discussed above). *Nibea leptolepis* (Ogilby 1918), another species distributed along coasts of northern Australia, might also belong to *Austronibea*.

Distribution: Northern and northwestern Australia and southern New Guinea

Genus *Nibea* Jordan and Thompson 1911, new definition

Type species: *Nibea mitsukurii* (Jordan and Snyder 1900)

Diagnosis: The revised genus includes only species of *Nibea* clade A according to our molecular phylogenetic results (Figs 2 and 3) and in Lo et al. [24]. Diagnosis for the revised *Nibea*, generally follows the original diagnoses of *Nibea* (swimbladder carrot-shaped or fan-like without well-developed dorsal limbs and with a number of branched appendages along its entire lateral side; the first appendage not extending to lateral face of pectoral arch; anterior pair of swimbladder appendages entering into head beyond transverse septum and branching between skull and upper gill arches; sulcus head of sagitta not very oblique, shape of sulcus tail variable but not deepened as a hollow cone; scales on body ctenoid; second anal-fin spine length 38 to 64% of head length except for *N*. *semifasciata*; no mental barbel) with some modifications, e.g., in having terminal (and generally large) or slightly inferior mouths and generally enlarged inner lower jaw teeth, which distinguish them from *Nibea* B (*Austronibea*) species.

Composition: The redefined genus *Nibea* contains seven currently recognized species: *N*. *albiflora* (Richardson 1846), *N*. *chui* Trewavas 1971, *N*. *coibor* (Hamilton 1822), *N*. *maculata* (Bloch and Schneider 1801), *N*. *mitsukurii*, *N*. *semifasciata* Chu, Lo & Wu 1963, and *N*. *soldado* (Lacepède 1802). The revised genus excludes *N*. *squamosa*, *N*. *microgenys*, and possibly *N*. *leptolepis*, all assigned here to *Austronibea*. *N*. *soldado* is a species complex that contains multiple inferred species (see further discussion below). The taxonomy of *N*. *maculata* requires a further investigation.

Distribution: Eastern Indian Ocean, Western Pacific, and Northwest Pacific.

### Species delimitation

The compiled *COI* dataset comprising the sequence data from 51 currently recognized sciaenid species for 308 samples (S1 Table) were also used for the species delimitation analyses with ABGD and GMYC tools. The ABGD analysis conducted by partitioning all the individuals with prior maximal distance (P) = 0.0324 results in 50 OTUs by both distance metrics, JC69 and K2P. However, the GMYC analysis determined a higher number, 95 OTUs with the single-threshold GMYC for the transition between coalescence and speciation branching patterns of -1.94618 (S3 Fig). Twenty-five common OTUs resulting from these two analyses were identified and considered here as robust support for the inferred species. Here, the validity of currently recognized species of *Bahaba taipingensis*, *Panna microlepis*, “*Larimichthys”* (= *Pseudolarimichthys*) *terengganui*, *Pterotolithus maculatus*, *Nibea mitsukurii*, *N*. *maculata*, “*Nibea”* (= *Austronibea*) *squamosa*, “*N*.*”* (= *Austronibea*) *microgenys*, *Austronibea oedogenys*, *Daysciaena albida*, *Johnius macropterus*, *J*. *majan* (another recently discovered new species) [29], *J*. *dussumieri*, *J*. *macrorhynus*, *J*. *novaeguineae*, *J*. *trachycephalus*, *J*. *trewavasae* and *J*. *carouna* is confirmed. Moreover, a few common OTUs (i.e., supported inferred species) are found within the recognized species (e.g., *Otolithes ruber*), indicating that these “species” contain cryptic or potential new species (Fig 2 and S3 Fig); two others (*J*. *borneensis* and *J*. *belangerii*) were found to be polyphyletic, further supporting the invalidity of these two species or potential errors in species identification in GenBank sequences (Fig 2 and S3 Fig).

In general, GMYC analysis tends to split more OTUs than the ABGD analysis does [4, 67–70]. Their different algorithms applied to the analyses can explain this incongruence. In fact, GMYC implements a strong theoretical basis that is underpinned by the transition from coalescent to speciation determined based on the inferred ultrametric gene (or time) tree; the variability in rates of genetic change as well the speciation rate and effective population size might affect the result of species delimitation [67, 70]. To avoid overestimating the species diversity suggested by the GMYC results, we firstly resolved the incongruent OTUs found between the two methods by merging the split sister GMYC OTUs and paraphyletic GMYC OTUs to a single one when no evidence of sufficient genetic divergence among samples (K2P < 3.2% in *COI*) and non-monophyly in the separate gene trees was observed (Fig 2). Based on this analysis, 14 revised OTUs were determined. These OTUs also match and confirm the following morpho-species: *Miichthys miiuy*, *Collichthys niveatus*, *C*. *lucidus*, *Larimichthys crocea*, *L*. *polyactis*, *Pennahia ovata*, *P*. *anea*, *P*. *pawak*, *P*. *argentata*, *P*. *macrocephalus*, *Nibea albiflora*, *Chrysochir aureus*, *Dendrophysa russelii* and *Johnius distinctus* (Fig 2).

In the next step for treating the incongruent OTUs, we merged the allopatric sister OTUs (when there was no significant difference in their morphology and no other evidence supporting their reproductive isolation) and separate the sympatric pairs. As a result, 60 species in total are inferred within the IWP sciaenid clade, of which 32 match the current definition of species (Fig 2).

### Cryptic diversity and necessity for further taxonomic consideration of species within the IWP clade

#### Atrobucca

From our species delimitation and phylogenetic analyses, two *Atrobucca* species were examined. Both ABGD and GMYC analyses recognized *A*. *nibe* CN211 collected in Kaohsiung, Taiwan as an inferred species, which is further confirmed by topology of individual gene and combined data trees. While ABGD clustered all other samples as a single OTU, GMYC split them into four (Fig 2 and S3 Fig).

According to the identification key proposed by Sasaki and Kailola [71], the difference in ratio of gill filament to standard length can separate *A*. *trewavasae* and *A*. *alcocki* (4.5–6.4%) from other *Atrobucca* spp. (2.4–3.5%) (e.g., *A*. *nibe*). For the sample of *A*. cf. *trewavasae* WJC6390 (collected from Dubai), its ratio calculated is 3.9%, which does not overlap with that of any known *Atrobucca* species. In *RAG1* gene tree, the sequence of *A*. cf. *trewavasae* WJC6390 diverges from other *Atrobucca* samples (S2 Fig). Based on these observations, it is likely that *A*. cf. *trewavasae* WJC6390 might represent a new *Atrobucca* species awaiting formal description.

In our other sampled *Atrobucca* specimens (excluding *A*. *nibe* CN211 [no voucher specimen kept] and *A*. *nibe* JF492920 [GenBank sequence; no specimen for examination]), the ratio of gill filament to standard length calculated falls into the range of 2.4–3.5% and the use of other morphological features diagnose our samples to be *A*. *nibe*. However, when the ratio of gill raker to gill filament of these *A*. *nibe* samples is calculated, we can observe differences between the individuals collected exclusively from eastern Taiwan (ratio = 0.88–1.03) and those collected from several localities in Taiwan (including eastern Taiwan) and in China (ratio = 1.37–1.8). While the *COI* K2P distances calculated among these samples are lower than 3.2% (0–2.2%), GMYC analysis delimits them as two OTUs that correspond to the observed morphological difference (Fig 2). In the *RAG1* gene tree, the corresponding samples from these two OTUs form two separate clades (S2 Fig). Moreover, we also found that the individuals of these OTUs co-occur in eastern Taiwan. Here, the combined evidence indicates that they could represent two reproductively isolated species.

#### Boesemania

*Boesemania* is the only freshwater sciaenid genus from the IWP realm and is currently considered to be monotypic. The present study analyzed two samples identified as *B*. *microlepis* and the evidence from all analyses suggests that they belong to two different species (Fig 2). The *COI* sequence of LC064301 was retrieved from GenBank and has been reported by Pringgenis and Susilowati [72]. It was caught in a non-freshwater habitat in Malacca Strait, Sumatra [72]. The *COI* sequence of KP722706 was obtained from an inland water sample collected in Thailand and was included in the analysis in Lo et al. [24]. The swimbladder of *B*. *microlepis* described by Pringgenis and Susilowati [72] is hammer-shaped without numerous appendages, which is not found in typical *B*. *microlepis*. Based on these observations (including the habitat preference), it is very likely that the atypical *B*. *microlepis* LC064301 is a new species waiting for formal descriptions.

#### Otolithes

The present study included all available sequence data from the two known tigertooth croakers of the genus *Otolithes*: 28 *COI* sequences of *O*. *ruber* (19 newly obtained in the present study with nine others retrieved from online databases) and one *COI* sequence of *O*. *cuvieri* (retrieved from GenBank). *Otolithes ruber* is a widespread species while *O*. *cuvieri* is restricted to the coasts of India and Pakistan in the Indian Ocean. Our combined evidence deduced from both available molecular data and morphological examination (see below) suggest that this genus contains five putative species (Fig 2). One is widely distributed in the IWP (assigned herein as IWP species); four others are locally distributed (e.g., in Bali; Bali species) or regionally distributed (e.g., in the Indian Ocean: West IO I, West IO II, and IO + SAS spp.).

The Bali species (WJC2779) is robustly supported by both ABGD and GMYC species delimitation results (Fig 2). This species is found to be sister to the clade containing exclusively samples from western Indian Ocean that are separated into two OTUs (putative species; i.e. West IO I sp. and West IO II sp.) with significant sequence divergences between them (*COI* K2P = 6.8–7%). In the *RAG1* gene tree, the Bali species is deeply divergent from others (S2 Fig).

For the single *O*. *cuvieri* individual sampled in this study, our analyses revealed that its *COI* sequence (from Maharashtra, western coast of India; retrieved from GenBank, FJ347924) is almost identical to that of *O*. *ruber* WJC1929 (from Northern Territory, Australia) and *O*. *ruber* (from Queensland, Australia; retrieved from GenBank, DQ107812). These three sequences cluster with two other *COI* sequences of *O*. *ruber* from the western Indian Ocean (their sequence divergences estimated from *COI* K2P distance are from 0 to 2.1%) (Fig 2 and S1 Fig). As the currently known *O*. *cuvieri* are only reported in the western Indian Ocean (along the coasts of India and Pakistan) [12], the fact that *O*. *cuvieri* FJ347924 was found nested within the IO + SAS species clade in our phylogenetic tree indicates a likely misidentification for this specimen. The other possibility could be that the IO + SAS sp. is actually *O*. *cuvieri* and the presence of “*O*. *cuvieri”* in Australian waters indicates a considerable range extension of the species. Further taxonomic investigation is necessary.

The results from the morphological examination of currently available specimens from Bali sp. (n = 1), West IO II sp. (n = 3), and IWP sp. (n = 10), show that West IO II sp. differs from the IWP sp. by having a reduced number of dorsal fin spines (IX vs. X) but a higher number of dorsal soft fin rays (30 vs. 27–29). In addition, more gill rakers were found on both upper and lower limbs in the West IO II sp. than those found in two other inferred species (5 and 12, respectively in West IO II sp.; 3–4 and 9–10, respectively in Bali sp. and IWP sp.). The swimbladder morphology of the two previously recognized *Otolithes* species [32] is characterized by a carrot-shaped swimbladder with 28 appendages or more (28 in *O*. *cuvieri* and 32–36 in *O*. *ruber*). All of our “*O*. *ruber*” specimens examined in this study have a carrot-shaped swimbladder, but with more than 35 pairs of fan-like appendages (37 in Bali sp.; 36–40 in IWP sp.). All the above evidence indicates that *O*. *ruber* contains cryptic and potential new species and the taxonomy of this species requires further investigation.

#### *Protonibea* and *Megalonibea*

*Protonibea* and *Megalonibea* are another two ‘monotypic’ sciaenid genera within the IWP clade. Our phylogenetic results show *Megalonibea* is nested within the *Protonibea diacanthus* clade. This renders *Protonibea* paraphyletic (Figs 2 and 3). In the species delimitation analyses based on our *COI* dataset, we included 15 samples from *P*. *diacanthus* and one from *M*. *fusca*. ABGD resolved them as a single OTU while GYMC suggested five (Fig 2 and S3 Fig). Whatever the results, all the analyses converge to conclude that *M*. *fusca*, a large-size sciaenid distributed in the East China Sea, is invalid (Fig 2). Interestingly, in a previous study [73], a comparison of the morphology of sagittas (the largest otoliths) of the large-size sciaenids (including *P*. *diacanthus*) found in archeological sites and those taken from fresh specimens of different sizes purchased at local fish markets in Pakistan, with that of *M*. *fusca* described in the literature, hypothesized that *M*. *fusca* are the large-size adult of *P*. *diacanthus*. Our study also supports this hypothesis and further reveals that *P*. *diacanthus/M*. *fusca* is a species complex that contains multiple putative species (Fig 2).

Based on our *COI* results, we merged the two GMYC OTUs comprising the sequences from the samples of *P*. *diacanthus* and *M*. *fusca* collected from the South China Sea and adjusted regions (assigned as SCS + CT) into a single one because of high similarity among their sequences (K2P = 0–0.5%; divergences lower than 3.2%; shared *RAG* 1 sequence types). The Gujarat OTU, Mumbai + Australian OTU and south Indian OTU, are considered as separated based on the criteria of sequence divergence (*COI* K2P = 3.3–4.5%), reciprocal monophyly inferred in the *COI* gene tree as well as their geographic distribution (sympatric distribution in the Indian Ocean) (Fig 2). In summary, four species are tentatively inferred within the genus *Protonibea* (here *Megalonibea* is considered to be invalid), but detail morphological and genetic examinations are needed to clarify the species’ status.

#### Nibea soldado

Even though most of the taxonomic status within the genus “*Nibea”* has been resolved ([24] and this study), some issues remain to be clarified at the species level. Our phylogenetic analyses find *N*. *soldado* to be non-monophyletic because the samples from other species (*N*. *chui* and *N*. *coibor*) are included in the “*N*. *soldado*” clade, which is supported by a high bootstrap value (BS = 95% in *COI* tree; BS = 80% in combined tree) (Figs 2 and 3). From our results of species delimitation based on the *COI* dataset, this clade contains multiple OTUs from which one (*N*. cf. *soldado* assigned as Nsol I sp.) is robustly supported by both of ABGD and GMYC analyses and the phylogenetic analyses (Figs 2 and 3, S2 Fig). Based on the sequence divergences estimated by *COI* K2P distance (3.2–14%), we can differentiate the remaining samples into three more OTUs (Nsol II to IV spp.); Nsol II contains the individuals collected from SAS; Nsol III sp. includes *N*. *chui* (GenBank accession no. KP722744), *N*. *coibor* (GenBank accession no. KM233452) and an undetermined sample (*N*. sp. WJC1745) collected from Vietnam (Fig 2); Nsol IV sp. comprises widespread individuals (from IO + SCS + CT). The *COI* sequences of the Nsol III samples are almost identical (K2P = 0–0.2%). While the same four species (Nsol I–IV spp.) are resolved in the combined gene analysis (Fig 3), Nsol III and IV samples cluster together into a single clade in the *RAG1* gene tree (S2 Fig).

Here, our morphological examination from available specimens, including the syntype of *N*. *soldado* (MNHN 7606; skin only), reveals that the specimens of Nsol III sp. (n = 2) are different from the other inferred “*N*. *soldado*” species (Nsol I, n = 10; Nsol IV, n = 8) and the syntype by having a lower number of dorsal fin soft rays (26–27 in Nsol III; 28–29 in Nsol I; 28–30 in Nsol IV; 29 in the syntype and 27–33 according to the literature [32, 74, 75]) and a lower number of outer gill rakers on first arch from both upper (3 in Nsol III; 4–5 in Nsol I; 4 in Nsol IV; no data from the syntype and the literature [32, 74, 75]) and lower limbs (7 in Nsol III; 8–11 in Nsol I; 9–10 in Nsol IV; no data from the syntype but 7–11 according to the literature [32, 74, 75]). Thus, we consider that Nsol III sp. is different from typical *N*. *soldado*.

In addition, Nsol III sp. also differs from *N*. *chui* and *N*. *coibor* in morphology and meristic counts (e.g., 24–25 of dorsal fin soft rays in *N*. *chui*). Based on our results, we suggest that Nsol III sp. likely represents a new *Nibea* species, and the GenBank sequences of *N*. *chui* (KP722744) and *N*. *coibor* (KM233452) included in Nsol III clade probably represent misidentification of samples.

Finally, the specimens from Nsol I sp. and Nsol IV sp. are morphologically similar to each other and are diagnosed to be *N*. *soldado* based on Sasaki’s identification key [32]. Their ratios of lower jaw length to head length are measured to be 31.2–35.1% in Nsol I and 30–37.1% in Nsol IV, respectively; yet, they are different from the description of *N*. *soldado* in the literature (44 to 53%). Overall, our study indicates that *N*. *soldado* also contains cryptic and potential new species, and the taxonomic status of this nominal species requires further clarification.

#### *Johnius* species

*Johnius* is the most diverse sciaenid genus whose monophyly has been confirmed [24; this study]. However, identification of species is challenging because of similarity in their external morphology and overlapping meristic counts [32].

A total of 88 *Johnius COI* sequences from “16” collected species (S1 Table) were included in the analyses. Without any *a priori* species hypothesis, based on the analyses of the *COI* data compiled in this study, we were able to determine 11 robustly supported OTUs (by both ABGD and GMYC analyses), and seven others that were further resolved by other data (genetic similarity, morphology, geography, etc.) (Fig 2). The obtained results led us to confirm the species status of *Johnius macropterus*, *J*. *majan*, *J*. *dussumieri*, *J*. *distinctus*, *J*. *carouna*, *J*. *macrorhynus*, *J*. *trachycephalus*, *J*. *novaeguineae* and *J*. *trewavasae*, and to formulate our suspicion of some taxonomic confusion within the genus.

*Johnius fuscolineatus* is often identified as *J*. *amblycephalus* as they are the only two *Johnius* species with stiff chin barbel, and co-occur in the Indian Ocean. Previous studies once suggested that *J*. *fuscolineatus* was a synonym of *J*. *amblycephalus* [65, 76]. However, Sasaki [77] reconsidered it as a valid species. In the *COI* gene tree, *J*. *fuscolineatus* is nested within *J*. *amblycephalus* (S1 Fig). The results from advanced species delimitation analyses show that *J*. *fuscolineatus* HQ945878 should belong to the GMYC determined OTU that also contains *J*. *amblycephalus* samples collected from the Indian Ocean (Dubai in Persian Gulf) (Fig 2). This OTU can be separated from the other identified *J*. *amblycephalus* OTUs by its differentiated genetic divergence (*COI* K2P = 5.2–11.1%; distinct *RAG1* sequences; S3 Fig). Additionally, its sympatric distribution with the sister OTU in Indian Ocean (*J*. *amblycephalus* May689 collected from Perlis of the west coast of Peninsular Malaysia) (Fig 2) further indicates that it is reproductively isolated from the other OTUs. We thus consider it as a putative species. However, the taxonomy of *J*. *fuscolineatus* needs to be further investigated when correctly identified specimens of *J*. *fuscolineatus* become available, as the only sequence for the species was retrieved from GenBank.

*Johnius borneensis* and *J*. *belangerii* represent another two problematic species within the genus, because they resolved here as polyphyletic groups (Figs 2 and 3). “*J*. *borneensis”* DQ107818 from Queensland, Australia appears to be an independent lineage to the *J*. *borneensis* widely sampled in the IWP (Fig 2). Unfortunately, the sequence of this particular taxon was retrieved from GenBank, and we were not able to determine if it was a species misidentification.

Our phylogenetic results resolved *J*. *belangerii* as two independent lineages (Fig 2). The *J*. *belangerii* EF607410 (GenBank sequence; sample collected from Guangdong, South China Sea) is grouped with our undetermined *Johnius* sample (May 181) collected from Penang, Malaysia. Based on the results of our species delimitation analyses from the *COI* dataset, both samples belong to the same putative species (Fig 2). The *J*. *belangerii* samples collected in the Indian Ocean (IO), East China Sea (ECS), South China Sea (SCS) and its adjacent region (Coral Triangle: CT) form a monophyletic group and a potential species is inferred according to the species delimitation results and other considerations (Fig 2). Finally, another *J*. *belangerii* sequence retrieved from GenBank (accession no. HQ711868) is found to be grouped with our sampled *J*. *heterolepis* (identified based on the FAO identification key) [32] and *J*. *grypotus* KC491206 (GenBank sequence) in the *COI* gene tree (Fig 2). Their sequences are very similar (*COI* K2P = 0.2–1.4%) and should be considered to be the same putative species (Fig 2). These results indicate that *J*. *grypotus* KC491206 and *J*. *belangerii* HQ711868 retrieved from GenBank are probably errors in species identification.

## Conclusions

Correct species taxonomy is necessary to study the phylogeny, biogeography, fisheries and conservation, and the combined molecular and morphological approach presented in this study assists with delimiting and predicting sciaenid species with some accuracy. From our analyses of 308 samples collected from different locations in the IWP for 51 sciaenid species, we infer a total of 60 species, of which 32 current morpho-species are confirmed. Cryptic and potential new species within *Atrobucca nibe*, *Otolithes ruber*, *Protonibea diacanthus*, *Nibea soldado* and *Johnius amblycephalus* were discovered. A new genus *Pseudolarimichthys* is herein proposed; *Nibea* and *Austronibea* are redefined. We also report problematic sequences deposited in public databases, likely due to misidentification of samples.

## Supporting information

S1 Fig Phylogenetic tree of the IWP sciaenids based on the *COI* gene inferred with the maximum-likelihood method using GTR+*Γ*+*I* model. Branch lengths are proportional to inferred nucleotide substitutions. Numbers at nodes represent bootstrap values in percentage. Values below 70% are not shown. * indicates 100% bootstrap support. Species name in bold indicates the individual might be misidentified. Black bars indicate the species delimitation results match the recognizing species while white bars indicate the monophyletic groups contains potential cryptic species. The tree is rooted with *Aplodinotus grunniens* according to Lo et al. [24]
(PDF)

S2 Fig Phylogenetic tree of the IWP sciaenids based on the *RAG1* gene inferred with the maximum-likelihood method using GTR+*Γ*+*I* model. Branch lengths are proportional to inferred nucleotide substitutions. Numbers at nodes represent bootstrap values in percentage. Values below 70% are not shown. * indicates 100% bootstrap support. Black bars indicate the species delimitation results match the recognizing species while white bars indicate the monophyletic groups contains potential cryptic species. The tree is rooted with *Aplodinotus grunniens* according to Lo et al. [24]
(PDF)

S3 Fig Bayesian inference of *COI* gene tree with delineated OTUs. White bars present the discordant results of both analyses while black bars indicate the consistent OTU clusters suggested by both delimitation analyses (i.e. robust results). Taxa name with different colors represent the geographical distribution of the samples; yellow: Indian Ocean (IO), green: South China Sea + Coral Triangle (SCS + CT), gray: Sahul Shelf and Australia (SAS) and blue: East China Sea (ECS).
(PDF)

S1 Table Taxa, gene and online databases numbers and the sample locality of representative species. The species name with cf. means that specimen is very similar and identified as that species. Asterisk shows the species inhabit freshwater. Sequences from GenBank are bold and underlined. ^o^ indicates the out-groups used in phylogenetic analysis.
(DOCX)

S2 Table Primers used in this study. Abbreviations of genes: *COI*, Cytochrome oxidase subunit I; *RAG 1*, Activating gene 1. Reverse primers in italics.
(DOCX)

S3 Table Descriptive statistics of sequences and phylogenetic performance from *COI* and *RAG1* dataset, respectively. (DOCX)

S4 Table Descriptive statistics of sequences and phylogenetic performance from each locus of combined dataset. (DOCX)
