# Correction: Molecular exploration of hidden diversity in the Indo-West Pacific sciaenid clade

**DOI:** 10.1371/journal.pone.0181511

**Published:** 2017-07-12

**Authors:** Pei-Chun Lo, Shu-Hui Liu, Siti Azizah Mohd Nor, Wei-Jen Chen

Within the Material and methods section, a subsection labeled “Nomenclatural acts” is missing. The Nomenclatural acts section should read as follows: The electronic edition of this article conforms to the requirements of the amended International Code of Zoological Nomenclature, and hence the new names contained herein are available under that Code from the electronic edition of this article. This published work and the nomenclatural acts it contains have been registered in ZooBank, the online registration system for the ICZN. The ZooBank LSIDs (Life Science Identifiers) can be resolved and the associated information viewed through any standard web browser by appending the LSID to the prefix “http://zoobank.org/”. The LSID for this publication is: urn:lsid:zoobank.org:pub: 6A246898-4ED6-4281-86BE-A4EB246C7493. The electronic edition of this work was published in a journal with an ISSN, and has been archived and is available from the following digital repositories: PubMed Central, LOCKSS, and Welt der Fische / World of Fishes (http://wp.worldfish.de).

Additionally, information is missing from the section titled “Higher-level taxonomic implications and revised classification.” The sixth paragraph should read as follows: Genus *Pseudolarimichthys* gen. nov. Lo, Liu, Mohd Nor and Chen urn:lsid:zoobank.org:act: A84C8F9D-FF00-46B7-8654-BCCD1B5628BF.
