# Supplementary figures and images for: Molecular exploration of hidden diversity in the Indo-West Pacific sciaenid clade
Source: PLoS One. 2017 Apr 28;12(4):e0176623. doi: 10.1371/journal.pone.0176623 (PMC5409148; doi:10.1371/journal.pone.0176623)

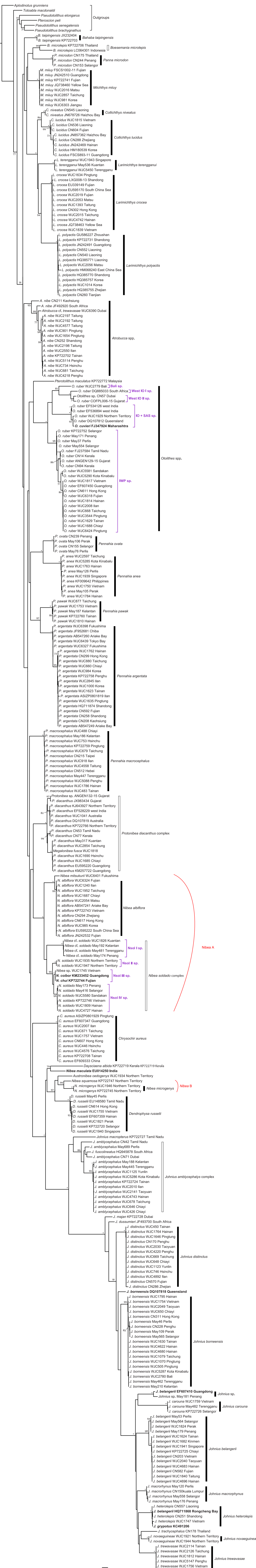

Supplement: S1 Fig — Branch lengths are proportional to inferred nucleotide substitutions. Numbers at nodes represent bootstrap values in percentage. Values below 70% are not shown. * indicates 100% bootstrap support. Species name in bold indicates the individual might be misidentified. Black bars indicate the species delimitation results match the recognizing species while white bars indicate the monophyletic groups contains potential cryptic species. The tree is rooted with Aplodinotus grunniens according to Lo et al. [24] (PDF) [file pone.0176623.s001.pdf]

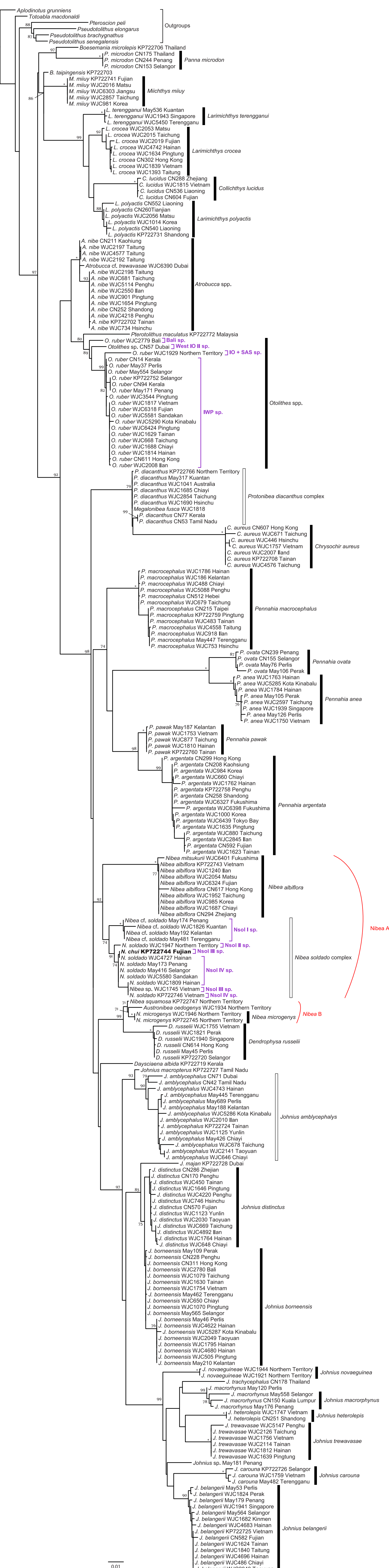

S2 Fig (Lo et al.)

Supplement: S2 Fig — Branch lengths are proportional to inferred nucleotide substitutions. Numbers at nodes represent bootstrap values in percentage. Values below 70% are not shown. * indicates 100% bootstrap support. Black bars indicate the species delimitation results match the recognizing species while white bars indicate the monophyletic groups contains potential cryptic species. The tree is rooted with Aplodinotus grunniens according to Lo et al. [24] (PDF) [file pone.0176623.s002.pdf]

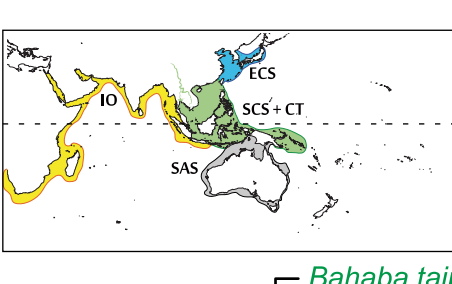

ABGD GMYC

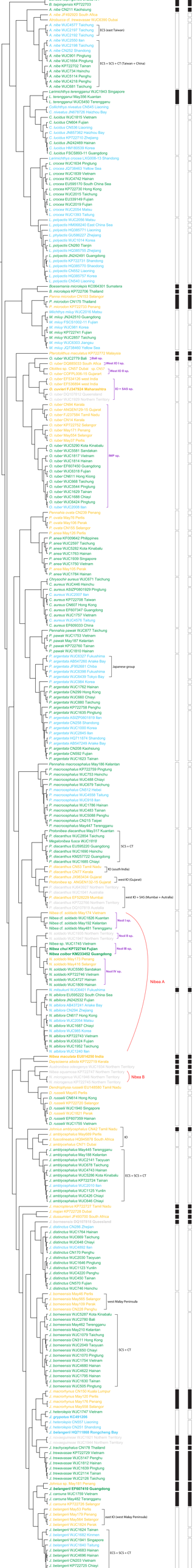

Supplement: S3 Fig — White bars present the discordant results of both analyses while black bars indicate the consistent OTU clusters suggested by both delimitation analyses (i.e. robust results). Taxa name with different colors represent the geographical distribution of the samples; yellow: Indian Ocean (IO), green: South China Sea + Coral Triangle (SCS + CT), gray: Sahul Shelf and Australia (SAS) and blue: East China Sea (ECS). (PDF) [file pone.0176623.s003.pdf]
