# Supplementary material for: Molecular exploration of hidden diversity in the Indo-West Pacific sciaenid clade
Source: PLoS One. 2017 Apr 28;12(4):e0176623. doi: 10.1371/journal.pone.0176623 (PMC5409148; doi:10.1371/journal.pone.0176623)
Supplement: S1 Table — The species name with cf. means that specimen is very similar and identified as that species. Asterisk shows the species inhabit freshwater. Sequences from GenBank are bold and underlined. o indicates the out-groups used in phylogenetic analysis. (DOCX) [file pone.0176623.s004.docx]

**S1 Table Taxa, gene and its accession number from online databases and the sample locality of representative species.** The species name with cf. means that specimen is very similar and identified as that species. Asterisk shows the species inhabit freshwater. Sequences from online databases (i.e. BOLD, GenBank and GRYOBANK) are bold and underlined. ^#^ indicated the specimen is conducted for the morphological measurements and counts of the major diagnostic characteristics. ^o^ indicates the species is used as outgroups in the analyses. ASIZP: Academia Sinica, Taipei, Taiwan; BIOUG<CAN>: Biodiversity Institute of Ontario, Guelph, Canada; MBCSC: Marine Biodiversity Collection of South China Sea, Chinese Academy of Sciences, Beijing, China; NBFGR: National Bureau of Fish Genetic Resources, Uttar Pradesh, India; NMMBP: National Museum of Marine Biology & Aquarium, Pingtung, Taiwan; NTUM: National Taiwan University Museums, Taipei, Taiwan.

| **Species Name** | **Tissue Sample ID** | **Specimen Voucher No.** | **GenBank Accession No.** | | **Sampling sites** |
| --- | --- | --- | --- | --- | --- |
|  |  |  | ***COI*** | ***RAG1*** |  |
| *Atrobucca nibe* | CN211 |  | KX777879 | KX777663 | Kaohsiung, Taiwan |
| *Atrobucca nibe* | CN252 |  | KX777880 | KX777664 | Shandong, China |
| *Atrobucca nibe* | WJC484 | NTUM11512 | **KP722702** | **KP722886** | An-Ping, Tainan, Taiwan |
| *Atrobucca nibe* | WJC681 | NTUM11537 | KX777881 | KX777665 | Wuchi, Taichung, Taiwan |
| *Atrobucca nibe* | WJC734 | NTUM11543 | KX777882 | KX777666 | Hsinchu, Taiwan |
| *Atrobucca nibe* | WJC901 |  | KX777883 | KX777667 | Xiao-Liu-Qiu, Pintung, Taiwan |
| *Atrobucca nibe* | WJC1654 |  | KX777884 | KX777668 | Pingtung, Taiwan |
| *Atrobucca nibe* | WJC2192 |  | KX777885 | KX777669 | Fugang, Taitung, Taiwan |
| *Atrobucca nibe* | WJC2197 |  | KX777886 | KX777670 | Fugang, Taitung, Taiwan |
| *Atrobucca nibe* | WJC2198 |  | KX777887 | KX777671 | Fugang, Taitung, Taiwan |
| *Atrobucca nibe* | WJC2550 |  | KX777888 | KX777672 | Dashi, Ilan, Taiwan |
| *Atrobucca nibe* | WJC4218 | NTUM11705 | KX777889 | KX777673 | Penghu, Taiwan |
| *Atrobucca nibe* | WJC4577 | NTUM11709 | KX777890 | KX777674 | Taitung, Taiwan |
| *Atrobucca nibe* | WJC5114 | NTUM11730 | KX777891 | KX777675 | Penghu, Taiwan |
| *Atrobucca nibe* |  | Smith 199.4 #4 | **JF492920** |  | Tugela Inshre, South Africa |
| *Atrobucca* cf. *trewavasae* | WJC6390 | NMMBP023017 | KX777892 | KX777676 | Deira, Dubai |
| *Austronibea oedogenys* | WJC1934 |  | KX777893 | **KP722887** | Northern Territory, Australia |
| *Bahaba taipingensis* | WJC1827 |  | **KP722703** | **KP722888** | Unknown |
| *Bahaba taipingensis* |  |  | **JX232404** |  | Unknown |
| **Boesemania microlepis* | CN177 |  | **KP722706** | **KP722890** | Thailand |
| **Boesemania microlepis* |  |  | **LC064301** |  | Sumatera, Indonesia |
| *Chrysochir aureus* | CN607 |  | KX777894 | KX777677 | Hong-Kong, China |
| *Chrysochir aureus* | WJC446 | NTUM11506 | KX777895 | KX777678 | Hsinchu, Taiwan |
| *Chrysochir aureus* | WJC451 | NTUM11510 | **KP722708** | **KP722892** | An-ping, Tainan, Taiwan |
| *Chrysochir aureus* | WJC671 | NTUM11532 | KX777896 | KX777679 | Wuchi, Taichung Taiwan |
| *Chrysochir aureus* | WJC1757 | NTUM11616 | KX777897 | KX777680 | HCM city, Vietnam |
| *Chrysochir aureus* | WJC2007 | NTUM11648 | KX777898 | KX777681 | Ilan, Taiwan |
| *Chrysochir aureus* | WJC4576 | NTUM11708 | KX777899 | KX777682 | Taitung, Taiwan |
| *Chrysochir aureus* | FSCS148-06 | MBCSC: Fish: GD9085078 | **EF607347** |  | Guangdong, China |
| *Chrysochir aureus* |  | ASIZP0801929 | **ASIZP0801929** |  | Pingtung, Taiwan |
| *Chrysochir aureus* |  | BW-A698 | **EF609333** |  | China |
| *Collichthys lucida* | CN288 |  | **KP722710** | **KP722894** | Zhejiang, China |
| *Collichthys lucidus* | CN536 |  | KX777900 | KX777683 | Liaoning, China |
| *Collichthys lucidus* | CN604 | NMMBP015217 | KX777901 | KX777684 | Fujian, China |
| *Collichthys lucidus* | WJC1815 | NTUM11634 | KX777902 | KX777685 | Vietnam |
| *Collichthys lucidus* | FSCS887-11 | MBCSC: Fish: XS116976 | **JN242469** |  | Hainan, China |
| *Collichthys lucidus* | FSCS893-11 | MBCSC: Fish: ZH1151861 | **JN242475** |  | Guangdong, China |
| *Collichthys lucidus* |  |  | **JN857362** |  | Haizhou Bay, China |
| *Collichthys lucidus* |  | NSMK: PI-000378 | **HM180539** |  | Korea |
| *Collichthys niveatus* | CN545 | NMMBP015213 | KX777903 |  | Liaoning, China |
| *Collichthys niveatus* |  |  | **JN678726** |  | Haizhou Bay, China |
| *Daysciaena albida* | CN92 |  | **KP722719** | **KP722903** | Kochi, Kerala, India |
| *Dendrophysa russelli* | CN154 |  | **KP722720** | **KP722904** | Selangor, Malaysia |
| *Dendrophysa russelii* | CN614 |  | KX777904 | KX777686 | Hong-Kong, China |
| *Dendrophysa russelii* | May45 | USM_CEMACS_045 | KX777905 | KX777687 | Kuala Perlis, Perlis, Malaysia |
| *Dendrophysa russelii* | WJC1755 | NTUM11614 | KX777906 | KX777688 | VanDon Town, Vietnam |
| *Dendrophysa russelii* | WJC1821 | NTUM11636 | KX777907 | KX777689 | Taiping, Perak, Malaysia |
| *Dendrophysa russelii* | WJC1940 |  | KX777908 | KX777690 | Pulau Utin, Singapore |
| *Dendrophysa russelii* | FSCS068-06 | MBCSC: Fish: GD 9086043 | **EF607359** |  | Hainan, China |
| *Dendrophysa russelii* |  | WL-M308 | **EU148580** |  | India |
| *Johnius* sp. | May181 | USM_CEMACS_181 | KX777922 | KX777704 | Penang, Malaysia |
| *Johnius amblycephalus* | CN42 |  | KX777909 | KX777691 | Chennai, Tamil Nadu, India |
| *Johnius amblycephalus* | CN71 |  | KX777910 | KX777692 | Dubai |
| *Johnius amblycephalus* | May188 | USM_CEMACS_188 | KX777911 | KX777693 | Kota Bharu, Kelantan, Malaysia |
| *Johnius amblycephalus* | May445 | USM_CEMACS_445 | KX777912 | KX777694 | Pulau Kambing, Terengganu, Malaysia |
| *Johnius amblycephalus* | May689 | NTUM11503 | KX777913 | KX777695 | Kuala Perlis, Perlis, Malaysia |
| *Johnius amblycephalus* | WJC426 |  | KX777914 | KX777696 | Budai, Chiayi, Taiwan |
| *Johnius amblycephalus* | WJC449 | NTUM11508 | **KP722724** | **KP722908** | An-ping, Tainan, Taiwan |
| *Johnius amblycephalus* | WJC646 | NTUM11517 | KX777915 | KX777697 | Chiayi, Taiwan |
| *Johnius amblycephalus* | WJC678 | NTUM11535 | KX777916 | KX777698 | Wuchi, Taichung, Taiwan |
| *Johnius amblycephalus* | WJC1125 |  | KX777917 | KX777699 | Yunlin, Taiwan |
| *Johnius amblycephalus* | WJC2010 | NTUM11651 | KX777918 | KX777700 | Ilan, Taiwan |
| *Johnius amblycephalus* | WJC2141 | NTUM11680 | KX777919 | KX777701 | Taoyuan |
| *Johnius amblycephalus* | WJC4743 | NTUM11725 | KX777920 | KX777702 | Sanya, Hainan, China |
| *Johnius amblycephalus* | WJC5286 | NTUM11733 | KX777921 | KX777703 | Kota Kinabalu, Sabah, Malaysia |
| *Johnius belangerii* | CN203 |  | KX777923 | KX777705 | Vietnam |
| *Johnius belangerii* | CN582 | NMMBP017642 | KX777924 | KX777706 | Fujian, China |
| *Johnius belangerii* | May53 | USM_CEMACS_053 | KX777925 | KX777707 | Kuala Perlis, Perlis, Malaysia |
| *Johnius belangerii* | May179 | USM_CEMACS_179 | KX777926 | KX777708 | Penang, Malaysia |
| *Johnius belangerii* | May564 | USM_CEMACS_564 | KX777927 | KX777709 | Sekinchan, Selangor, Malaysia |
| *Johnius belangerii* | WJC486 | NTUM11514 | **KP722725** | **KP722909** | Chiayi, Taiwan |
| *Johnius belangerii* | WJC1624 | NTUM11593 | KX777928 | KX777710 | An-ping, Tainan, Taiwan |
| *Johnius belangerii* | WJC1682 |  | KX777929 | KX777711 | Kinmen, Taiwan |
| *Johnius belangerii* | WJC1824 |  | KX777930 | KX777712 | Matang, Perak, Malaysia |
| *Johnius belangerii* | WJC1840 | NTUM11638 | KX777931 | KX777713 | Wuchi, Taichung, Taiwan |
| *Johnius belangerii* | WJC1941 |  | KX777932 | KX777714 | Pulau Utin, Singapore |
| *Johnius belangerii* | WJC2040 | NTUM11658 | KX777933 | KX777715 | Taoyuan, Taiwan |
| *Johnius belangerii* | WJC4683 | NTUM11712 | KX777934 | KX777716 | Ying Ge Hai, Hainan, China |
| *Johnius belangerii* | WJC4696 | NTUM11716 | KX777935 | KX777717 | Huang Liu, Hainan, China |
| *Johnius belangerii* | FSCS297-06 | MBCSC: Fish: GD 9087008 | **EF607410** |  | Guangdong, China |
| *Johnius belangerii* | IPCASFY_Jb_001 |  | **HQ711868** |  | Rongchen Bay, China |
| *Johnius borneensis* | CN228 |  | KX777936 | KX777718 | Penang, Malaysia |
| *Johnius borneensis* | CN311 |  | KX777937 | KX777719 | Hong-Kong, China |
| *Johnius borneensis* | May46 | USM_CEMACS_046 | KX777938 | KX777720 | Kuala Perlis, Perlis, Malaysia |
| *Johnius borneensis* | May109 | USM_CEMACS_109 | KX777939 | KX777721 | Lumut, Perak, Malaysia |
| *Johnius borneensis* | May210 | USM_CEMACS_210 | KX777940 | KX777722 | Bota Bharu, Kelantan, Malaysia |
| *Johnius borneensis* | May462 | USM_CEMACS_462 | KX777941 | KX777722 | Pulau Kambing, Terengganu, Malaysia |
| *Johnius borneensis* | May565 | USM_CEMACS_565 | KX777942 | KX777724 | Sekinchan, Selangor, Malaysia |
| *Johnius borneensis* | WJC505 | NTUM11516 | KX777943 | KX777725 | Pingtung, Taiwan |
| *Johnius borneensis* | WJC650 | NTUM11520 | KX777944 | KX777726 | Chiayi, Taiwan |
| *Johnius borneensis* | WJC1070 | NTUM11569 | KX777945 | KX777727 | Xiao-Liu-Qiu, Pintung, Taiwan |
| *Johnius borneensis* | WJC1079 | NTUM11575 | KX777946 | KX777728 | Wuchi, Taichung, Taiwan |
| *Johnius borneensis* | WJC1630 | NTUM11597 | KX777947 | KX777729 | An-ping, Tainan, Taiwan |
| *Johnius borneensis* | WJC1754 | NTUM11613 | KX777948 | KX777730 | VanDon Town, Vietnam |
| *Johnius borneensis* | WJC1795 | NTUM11627 | KX777949 | **KP722910** | Sanya, Hainan, China |
| *Johnius borneensis* | WJC2049 | NTUM11660 | KX777950 | KX777731 | Taoyuan, Taiwan |
| *Johnius borneensis* | WJC2780 | NTUM11697 | KX777951 | KX777732 | Jimbaran Bay, Bali |
| *Johnius borneensis* | WJC4622 | NTUM11720 | KX777952 | KX777733 | Huang Liu, Hainan, China |
| *Johnius borneensis* | WJC4680 |  | KX777953 | KX777734 | Ying Ge Hai, Hainan, China |
| *Johnius borneensis* | WJC5287 | NTUM11734 | KX777954 | KX777735 | Kota Kinabalu, Sabah, Malaysia |
| *Johnius borneensis* |  | BIOUG<CAN>: BW-A705 | **DQ107818** |  | Queensland, Australia |
| *Johnius carouna* | CN157 |  | **KP722726** | **KP722911** | Selangor, Malaysia |
| *Johnius carouna* | May482 | USM_CEMACS_482 | KX777955 | KX777736 | Pulau Kambing, Terengganu, Malaysia |
| *Johnius carouna* | WJC1759 | NTUM11618 | KX777956 | KX777737 | HCH city, Vietnam |
| *Johnius distinctus* | CN170 |  | KX777957 | KX777738 | Penghu, Taiwan |
| *Johnius distinctus* | CN286 |  | KX777958 | KX777739 | Zhejiang, China |
| *Johnius distinctus* | CN570 |  | KX777959 | KX777740 | Fujian, China |
| *Johnius distinctus* | WJC450 | NTUM11509 | KX777960 | **KP722912** | An-ping, Tainan, Taiwan |
| *Johnius distinctus* | WJC648 | NTUM11518 | KX777961 | KX777741 | Chiayi, Taiwan |
| *Johnius distinctus* | WJC669 | NTUM11530 | KX777962 | KX777742 | Wuchi, Taichung, Taiwan |
| *Johnius distinctus* | WJC746 | NTUM11744 | KX777963 | KX777743 | Hsinchu, Taiwan |
| *Johnius distinctus* | WJC1123 |  | KX777964 | KX777744 | Yunlin, Taiwan |
| *Johnius distinctus* | WJC1646 | NTUM11603 | KX777965 | KX777745 | Pingtung, Taiwan |
| *Johnius distinctus* | WJC1764 | NTUM11621 | KX777966 | KX777746 | Xincun, Hainan, China |
| *Johnius distinctus* | WJC2030 | NTUM11656 | KX777967 | KX777747 | Taoyuan, Taiwan |
| *Johnius distinctus* | WJC4220 | NTUM11706 | KX777968 | KX777748 | Penghu, Taiwan |
| *Johnius distinctus* | WJC4892 |  | KX777969 | KX777749 | Dashi, Ilan, Taiwan |
| *Johnius dussumieri* |  | Smith 199.6 #2 | **JF493700** |  | South Africa |
| *Johnius fuscolineatus* |  | ADC10_199.5 #7 | **HQ945878** |  | South Africa |
| *Johnius grypotus* |  |  | **KC491206** |  | Unknown |
| *Johnius heterolepis* | CN251 |  | KX777970 | KX777750 | Shandong, China |
| *Johnius heterolepis* | CN557 | NMMBP015211 | KX777971 |  | Liaoning, China |
| *Johnius heterolepis* | WJC1747 | NTUM11607 | KX777972 | KX777751 | Do Son Town, Vietnam |
| *Johnius macropterus* | CN38 |  | **KP722727** | **KP722914** | Chennai, Tamil Nadu, India |
| *Johnius macrorhynus* | CN150 |  | KX777973 | KX777752 | Kuala Lumpur, Malaysia |
| *Johnius macrorhynus* | May120 | USM_CEMACS_120 | KX777974 | KX777753 | Kuala Perlis, Perlis, Malaysia |
| *Johnius macrorhynus* | May176 | USM_CEMACS_176 | KX777975 | KX777754 | Penang, Malaysia |
| *Johnius macrorhynus* | May558 | USM_CEMACS_558 | KX777976 | KX777755 | Sekinchan, Selangor, Malaysia |
| *Johnius majan* | CN65 |  | **KP722728** | **KP722915** | Dubai |
| *Johnius novaeguineae* | WJC1921 |  | KX777977 | KX777756 | Northern Territory, Australia |
| *Johnius novaeguineae* | WJC1944 |  | KX777978 | KX777757 | Northern Territory, Australia |
| *Johnius trachycephalus* | CN178 |  | KX777979 | KX777758 | Thailand |
| *Johnius trewavasae* | WJC1639 | NTUM11600 | KX777980 | KX777759 | Pingtung, Taiwan |
| *Johnius trewavasae* | WJC1756 | NTUM11615 | **KP722729** | **KP722916** | Van Don Town, Vietnam |
| *Johnius trewavasae* | WJC1812 | NTUM11632 | KX777981 | KX777760 | Huang Liu, Hainan, Taiwan |
| *Johnius trewavasae* | WJC2114 | NTUM11668 | KX777982 | KX777761 | An-ping, Tainan, Taiwan |
| *Johnius trewavasae* | WJC2126 | NTUM11673 | KX777983 | KX777762 | Wuchi, Taichung, Taiwan |
| *Johnius trewavasae* | WJC5147 | NTUM11750 | KX777984 | KX777763 | Penghu, Taiwan |
| *Larimichthys crocea* | CN302 |  | **KP722730** | **KP722917** | Hong-Kong, China |
| *Larimichthys crocea* | WJC1393 | NTUM11586 | KX777985 | KX777764 | Taitung, Taiwan |
| *Larimichthys crocea* | WJC1634 |  | KX777986 | KX777765 | Pingtung, Taiwan |
| *Larimichthys crocea* | WJC1839 | NTUM11637 | KX777987 | KX777766 | Vietnam |
| *Larimichthys crocea* | WJC2015 | NTUM11653 | KX777988 | KX777767 | Wuchi, Taichung, Taiwan |
| *Larimichthys crocea* | WJC2019 | NTUM11654 | KX777989 | KX777768 | Xiamen, Fujian, China |
| *Larimichthys crocea* | WJC2053 | NTUM11663 | KX777990 | KX777769 | Matsu, Taiwan |
| *Larimichthys crocea* | WJC4742 | NTUM11724 | KX777991 | KX777770 | Sanya, Hainan, Taiwan |
| *Larimichthys crocea* | F00219 |  | **JQ738463** |  | Yellow Sea |
| *Larimichthys crocea* | FSCS353-07 | MBCSC: Fish: ZC I07057 | **EU595170** |  | South China Sea |
| *Larimichthys crocea* |  | ZJ104 | **LXG008-13** |  | Shandong, China |
| *Larimichthys crocea* |  |  | **EU339149** |  | Fujian, China |
| *Larimichthys polyactis* | CN260 |  | KX777992 | KX777771 | Tianjin, China |
| *Larimichthys polyactis* | CN270 | NMMBP017644 | **KP722731** | **KP722918** | Shandong, China |
| *Larimichthys polyactis* | CN540 | NMMBP015215 | KX777993 | KX777772 | Liaoning, China |
| *Larimichthys polyactis* | CN552 | NMMBP015219 | KX777994 | KX777773 | Liaoning, China |
| *Larimichthys polyactis* | WJC1014 |  | KX777995 | KX777774 | Yeosu, Korea |
| *Larimichthys polyactis* | WJC2056 | NTUM11665 | KX777996 | KX777775 | Matsu, Taiwan |
| *Larimichthys polyactis* | B4 |  | **HM068240** |  | East China Sea |
| *Larimichthys polyactis* | C25 |  | **HQ385770** |  | Shandong, China |
| *Larimichthys polyactis* | D30 |  | **HQ385771** |  | Liaoning, China |
| *Larimichthys polyactis* | FSCS972-11 | MBCSC: Fish: ZH1151878 | **JN242491** |  | Guangdong, China |
| *Larimichthys polyactis* | K1 |  | **HQ385757** |  | Korea |
| *Larimichthys polyactis* | Z27 |  | **HQ385755** |  | Zhejian, China |
| *Larimichthys polyactis* |  |  | **GU586227** |  | Zhejian, China |
| *Larimichthys terengganui* | May356 | USM_CEMACS_356 | KX777997 | KX777776 | Pahang, Kuantan, Malaysia |
| *Larimichthys terengganui* | WJC1943 |  | KX777998 | KX777777 | Singapore |
| *Larimichthys terengganui* | WJC5450 |  | KX777999 | KX777778 | Terengganu, Malaysia |
| *Megalonibea fusca* | WJC1818 |  | **KP722735** | **KP722922** | Unknown |
| *Miichthys miiuy* | WJC489 |  | **KP722741** | **KP722928** | Fuzhou, Fujian, China |
| *Miichthys miiuy* | WJC981 | NTUM11558 | KX778000 | KX777779 | Yeosu, Korea |
| *Miichthys miiuy* | WJC2016 |  | KX778001 | KX777780 | Matsu, Taiwan |
| *Miichthys miiuy* | WJC2857 |  | KX778002 | KX777781 | Wuchi, Taichung, Taiwan |
| *Miichthys miiuy* | WJC6303 | NTUM11735 | KX778003 | KX777782 | Jiangsu, China |
| *Miichthys miiuy* | F00211 |  | **JQ738460** |  | Guangdong, China |
| *Miichthys miiuy* | FSCS1002-11 | MBCSC: Fish: ZP1141244 | **JN242509** |  | Fujian, China |
| *Miichthys miiuy* | FSCS1003-11 | MBCSC: Fish: ZH11518151 | **JN242510** |  | Guangdong, China |
| *Nibea* sp. *^#^* | WJC1745 | NTUM11605 | KX778025 | KX777804 | Do Son Town, Vietnam |
| *Nibea albiflora* | CN294 |  | KX778004 | KX777783 | Zhejiang, China |
| *Nibea albiflora* | CN617 |  | KX778005 | KX777784 | Hong-Kong, China |
| *Nibea albiflora* | WJC985 | NTUM11561 | KX778006 | KX777785 | Yeosu, Korea |
| *Nibea albiflora* | WJC1240 | NTUM11579 | KX778007 | KX777786 | Ilan, Taiwan |
| *Nibea albiflora* | WJC1687 |  | KX778008 | KX777787 | Chiayi, Taiwan |
| *Nibea albiflora* | WJC1752 | NTUM11611 | **KP722743** | **KP722930** | QuangYen Town, Vietnam |
| *Nibea albiflora* | WJC1952 | NTUM11641 | KX778009 | KX777788 | Wuchi, Taichung, Taiwan |
| *Nibea albiflora* | WJC2054 | NTUM11664 | KX778010 | KX777789 | Matsu, Taiwan |
| *Nibea albiflora* | WJC6324 |  | KX778011 | KX777790 | Xiamen, Fujian, China |
| *Nibea albiflora* | FSCS1032-11 | MBCSC: Fish: ZH11518125 | **JN242532** |  | Fujian, China |
| *Nibea albiflora* | FSCS412-07 | MBCSC: Fish: ZC I07065 | **EU595222** |  | South China Sea |
| *Nibea albiflora* | Nial01 |  | **AB547241** |  | Ariake Bay, Japan |
| *Nibea chui^#^* | CN560 | NMMBP019364 | **KP722744** | KX777791 | Xiamen, Fujian, China |
| *Nibea coibor* |  |  | **KM233452** |  | Guangdong, China |
| *Nibea maculata* | WL-M303 |  | **EU014250** |  | India |
| *Nibea microgenys* | WJC1946 |  | KX778012 | KX777792 | Northern Territory, Australia |
| *Nibea microgenys* | WJC1951 |  | **KP722745** | **KP722931** | Bynoe Harbour |
| *Nibea mitsukurii* | WJC6401 |  | KX778013 | KX777793 | Off Ukedo, Fukushima, Japan |
| *Nibea soldado^#^* | May173 | USM_CEMACS_173 | KX778014 | KX777794 | Penang, Malaysia |
| *Nibea* cf. *soldado^#^* | May174 | USM_CEMACS_174 | KX778015 | KX777795 | Penang, Malaysia |
| *Nibea* cf. *soldado^#^* | May192 | USM_CEMACS_192 | KX778016 | KX777796 | Kota Bharu, Kelantan, Malaysia |
| *Nibea soldado^#^* | May416 | USM_CEMACS_416 | KX778017 | KX777797 | Kuala Selangor, Selangor, Malaysia |
| *Nibea* cf. *soldado^#^* | May481 | USM_CEMACS_481 | KX778018 | KX777798 | Pulau Kambing, Terengganu, Malaysia |
| *Nibea soldado^#^* | WJC1761 | NTUM11619 | **KP722746** | **KP722932** | HCH city, Vietnam |
| *Nibea soldado^#^* | WJC1809 | NTUM11630 | KX778019 | KX777799 | Huang Liu, Hainan, China |
| *Nibea* cf. *soldado* | WJC1826 |  | KX778020 | KX777800 | Kuantan, Malaysia |
| *Nibea soldado* | WJC1935 |  | KX778021 |  | Northern Territory, Australia |
| *Nibea soldado* | WJC1947 |  | KX778022 | KX777801 | Northern Territory, Australia |
| *Nibea soldado^#^* | WJC4727 | NTUM11723 | KX778023 | KX777802 | Sanya, Hainan, China |
| *Nibea soldado* | WJC5580 |  | KX778024 | KX777803 | Sandakan, Sabah, Malaysia |
| *Nibea squamosa* | WJC1945 |  | **KP722747** | **KP722933** | Northern Territory, Australia |
| *Otolithes* sp. *^#^* | CN57 | NMMBP019364 | KX778026 | KX777805 | Abu Dhabi, United Arab |
| *Otolithes* *cuvieri* |  | NBFGR: JC256 | **FJ347924** |  | Maharachtra, India |
| *Otolithes ruber* | CN14 |  | KX778027 | KX777806 | Kerala, India |
| *Otolithes ruber* | CN94 |  | KX778028 | KX777807 | Kochi, Kerala, India |
| *Otolithes ruber* | CN161 |  | **KP722752** | **KP722936** | Malaysia |
| *Otolithes ruber* | CN611 |  | KX778029 | KX777808 | Hong-Kong. China |
| *Otolithes ruber^#^* | May37 | USM_CEMACS_037 | KX778030 | KX777809 | Kuala Perlis, Perlis, Malaysia |
| *Otolithes ruber^#^* | May171 | USM_CEMACS_171 | KX778031 | KX777810 | Penang, Malaysia |
| *Otolithes ruber^#^* | May554 | USM_CEMACS_554 | KX778032 | KX777811 | Sekinchan, Selangor, Malaysia |
| *Otolithes ruber^#^* | WJC668 | NTUM11529 | KX778033 | KX777812 | Wuchi, Taichung, Taiwan |
| *Otolithes ruber^#^* | WJC1629 | NTUM11596 | KX778034 | KX777813 | An-ping, Tainan, Taiwan |
| *Otolithes ruber* | WJC1688 |  | KX778035 | KX777814 | Chiayi, Taiwan |
| *Otolithes ruber^#^* | WJC1814 | NTUM11633 | KX778036 | KX777815 | Huang Liu, Hainan, China |
| *Otolithes ruber^#^* | WJC1817 | NTUM11635 | KX778037 | KX777816 | Vietnam |
| *Otolithes ruber* | WJC1929 |  | KX778038 | KX777817 | Northern Territory, Australia |
| *Otolithes ruber^#^* | WJC2008 | NTUM11649 | KX778039 | KX777818 | Ilan, Taiwan |
| *Otolithes ruber^#^* | WJC2779 | NTUM11696 | KX778040 | KX777819 | Jimbaran Bay, Bali |
| *Otolithes ruber* | WJC3544 |  | KX778041 | KX777820 | Donggang, Pingtung, Taiwan |
| *Otolithes ruber* | WJC5290 |  | KX778042 | KX777821 | Kota Kinabalu, Sabah, Malaysia |
| *Otolithes ruber* | WJC5581 |  | KX778043 | KX777822 | Sandakan, Sabah, Malaysia |
| *Otolithes ruber* | WJC6318 |  | KX778044 | KX777823 | Xiamen, Fujian, China |
| *Otolithes ruber^#^* | WJC6424 | NTUM11753 | KX778045 | KX777824 | Pingtung, Taiwan |
| *Otolithes ruber* | A |  | **EF534126** |  | India |
| *Otolithes ruber* | FSCS313-06 | MBCSC: Fish: GD 9087017 | **EF607450** |  | Guangdong, China |
| *Otolithes ruber* | JAUCoFAquaDNA_21 |  | **COFPL006-15** |  | Gujarat, India |
| *Otolithes ruber* | LQDWL-MP1415-FISH-57 |  | **ANGEN129-15** |  | Gujarat, India |
| *Otolithes ruber* | NBFGR: 1138b |  | **FJ237584** |  | Tamil Nadu, India |
| *Otolithes ruber* |  | BIOUG<CAN>: BW-A699 | **DQ107812** |  | Queensland, Australia |
| *Otolithes ruber* |  | Smith 199.7 #5 | **DQ885033** |  | South Africa |
| *Otolithes ruber* |  | WSLOR-B | **EF536894** |  | India |
| *Panna microdon* | CN153 |  | KX778046 | KX777825 | Selangor, Malaysia |
| *Panna microdon* | CN175 |  | KX778047 | KX777826 | Thailand |
| *Panna microdon* | CN244 |  | **KP722755** | **KP722939** | Penang, Malaysia |
| *Pennahia anea* | May105 | USM_CEMACS_105 | KX778048 | KX777827 | Lumut, Perak, Malaysia |
| *Pennahia anea* | May126 | USM_CEMACS_126 | KX778049 | KX777828 | Kuala Perlis, Perlis, Malaysia |
| *Pennahia anea* | WJC1750 | NTUM11610 | KX778050 | KX777829 | Quang Yen Town |
| *Pennahia anea* | WJC1763 |  | KX778051 | KX777830 | Hainan, China |
| *Pennahia anea* | WJC1784 | NTUM11623 | KX778052 | KX777831 | Sanya, Hainan, China |
| *Pennahia anea* | WJC1939 |  | KX778053 | KX777832 | Pulau Utin, Singapore |
| *Pennahia anea* | WJC2597 | NTUM11685 | KX778054 | KX777833 | Wuchi, Taichung, Taiwan |
| *Pennahia anea* | WJC5282 | NTUM11732 | KX778055 | KX777834 | Kota Kinabalu, Sabah, Malaysia |
| *Pennahia anea* |  | ARO 167 | **KF009642** |  | Philippines |
| *Pennahia argentata* | CN172 |  | **KP722758** | **KP722941** | Penghu, Taiwan |
| *Pennahia argentata* | CN208 |  | KX778056 | KX777835 | Kaohsiung, Taiwan |
| *Pennahia argentata* | CN258 |  | KX778057 | KX777836 | Shandong, China |
| *Pennahia argentata* | CN299 |  | KX778058 | KX777837 | Hong-Kong, China |
| *Pennahia argentata* | CN592 |  | KX778059 | KX777838 | Xiamen, Fujian, China |
| *Pennahia argentata* | WJC660 | NTUM11524 | KX778060 | KX777839 | Chiayi, Taiwan |
| *Pennahia argentata* | WJC880 | NTUM11551 | KX778061 | KX777840 | Wuchi, Taichung, Taiwan |
| *Pennahia argentata* | WJC984 | NTUM11560 | KX778062 | KX777841 | Jagalchi, Korea |
| *Pennahia argentata* | WJC1000 |  | KX778063 | KX777842 | Yeosu, Korea |
| *Pennahia argentata* | WJC1623 |  | KX778064 | KX777843 | An-ping, Tainan, Taiwan |
| *Pennahia argentata* | WJC1635 | NTUM11599 | KX778065 | KX777844 | Pingtung, Taiwan |
| *Pennahia argentata* | WJC1762 | NTUM11620 | KX778066 | KX777845 | Hainan, China |
| *Pennahia argentata* | WJC2845 |  | KX778067 | KX777846 | Ilan, Taiwan |
| *Pennahia argentata* | WJC6327 |  | KX778068 | KX777847 | Off Ukedo, Fukushima, Japan |
| *Pennahia argentata* | WJC6398 |  | KX778069 | KX777848 | Off Hirono, Fukushima, Japna |
| *Pennahia argentata* | WJC6439 | NTUM11740 | KX778070 | KX777849 | Tokyo Bay, Japan |
| *Pennahia argentata* | Pear06 |  | **AB547249** |  | Ariake Bay, Japan |
| *Pennahia argentata* | Pear17 |  | **AB547260** |  | Ariake Bay, Japan |
| *Pennahia argentata* |  | IOCASFY_Pa_001 | **HQ711874** |  | Shandong, China |
| *Pennahia argentata* |  | SIRG1 | **JF952681** |  | Chiba, Japan |
| *Pennahia argentata* |  | ASIZP0801819 | **ASIZP0801819** |  | Ilan, Taiwan |
| *Pennahia macrocephalus* | CN215 |  | KX778071 | KX777850 | Taipei, Taiwan |
| *Pennahia macrocephalus* | CN512 | NMMBP017646 | KX778072 | KX777851 | Hebei, China |
| *Pennahia macrocephalus* | CN640 |  | **KP722759** | **KP722942** | Pingtung, Taiwan |
| *Pennahia macrocephalus* | May186 | USM_CEMACS_186 | KX778073 | KX777852 | Kota Bharu, Kelantan, Malaysia |
| *Pennahia macrocephalus* | May447 | USM_CEMACS_447 | KX778074 | KX777853 | Pulau Kambing, Terengganu, Malaysia |
| *Pennahia macrocephalus* | WJC483 | NTUM11511 | KX778075 | KX777854 | An-ping, Tainan, Taiwan |
| *Pennahia macrocephalus* | WJC488 | NTUM11515 | KX778076 | KX777855 | Chiayi, Taiwan |
| *Pennahia macrocephalus* | WJC679 | NTUM11536 | KX778077 | KX777856 | Wuchi, Taichung, Taiwan |
| *Pennahia macrocephalus* | WJC754 | NTUM11544 | KX778078 | KX777857 | Hsinchu, Taiwan |
| *Pennahia macrocephalus* | WJC918 | NTUM11554 | KX778079 | KX777858 | Dashi, Ilan, Taiwan |
| *Pennahia macrocephalus* | WJC1786 | NTUM11625 | KX778080 | KX777859 | Sanya, Hainan, Chia |
| *Pennahia macrocephalus* | WJC4558 | NTUM11707 | KX778081 | KX777860 | Taitung, Taiwan |
| *Pennahia macrocephalus* | WJC5088 | NTUM11729 | KX778082 | KX777861 | Penghu, Taiwan |
| *Pennahia ovata* | CN155 |  | KX778083 | KX777862 | Selangor, Malaysia |
| *Pennahia ovata* | CN239 |  | KX778084 | KX777863 | Penang, Malaysia |
| *Pennahia ovata* | May76 | USM_CEMACS_076 | KX778085 | KX777864 | Kuala Perlis, Perlis, Malaysia |
| *Pennahia ovata* | May106 | USM_CEMACS_106 | KX778086 | KX777865 | Lumut, Perak, Malaysia |
| *Pennahia pawak* | May187 | USM_CEMACS_187 | KX778087 | KX777866 | Kota Bharu, Kelantan, Malaysia |
| *Pennahia pawak* | WJC877 | NTUM11549 | KX778088 | KX777867 | Wuchi, Taichung, Taiwan |
| *Pennahia pawak* | WJC448 | NTUM11507 | **KP722760** | **KP722943** | An-ping, Tainan, Taiwan |
| *Pennahia pawak* | WJC1753 | NTUM11612 | KX778089 | KX777868 | Van Don Town, Vietnam |
| *Pennahia pawak* | WJC1810 | NTUM11631 | KX778090 | KX777869 | Huang Liu, Hainan, China |
| *Protonibea diacanthus* | CN53 |  | KX778091 | KX777870 | Chennai, Tamil Nadu, India |
| *Protonibea diacanthus* | CN77 |  | KX778092 | KX777871 | Kochi, Kerala, India |
| *Protonibea diacanthus* | May317 | USM_CEMACS_317 | KX778093 | KX777872 | Pahang, Kuantan, Malaysia |
| *Protonibea diacanthus* | WJC1041 |  | KX778094 | KX777873 | Australia |
| *Protonibea diacanthus* | WJC1685 |  | KX778095 | KX777874 | Chiayi, Taiwan |
| *Protonibea diacanthus* | WJC1690 | NTUM11604 | KX778096 | KX777875 | Hsinchu, Taiwan |
| *Protonibea diacanthus* | WJC1950 |  | **KP722766** | **KP722948** | Northern Territory, Australia |
| *Protonibea diacanthus* | WJC2854 | NTUM11699 | KX778097 | KX777876 | Wuchi, Taichung, Taiwan |
| *Protonibea diacanthus* | A |  | **EF528229** |  | North western Indian coast |
| *Protonibea diacanthus* | FSCS505-07 | MBCSC: Fish: ZC I07164 | **EU595220** |  | Guangdong, China |
| *Protonibea* sp. | LQDWL-MP1415-FISH-60 |  | **ANGEN132-15** |  | Gujarat, India |
| *Protonibea diacanthus* |  | BW-A690 | **DQ107819** |  | Australia |
| *Protonibea diacanthus* |  | NF48 | **JX983434** |  | Gujarat, India |
| *Protonibea diacanthus* |  |  | **KJ643927** |  | Northern Territory, Australia |
| *Protonibea diacanthus* |  |  | **KM257722** |  | Guangdong, China |
| *Pterotolithus maculatus* | CN165 |  | **KP722772** | **KP722953** | Malaysia |
| *Aplodinotus grunnies* ^o^ | WJC158 |  | **KP722699** | **KP722882** |  |
| *Pseudotolithus brachygnathus* ^o^ | WJC550A |  | **KP722767** | **KP722949** |  |
| *Pseudotolithus elongatus* ^o^ | WJC507A |  | **KP722768** | **KP722950** |  |
| *Pseudotolithus senegalensis* ^o^ | WJC508 |  | KX778098 | KX777877 |  |
| *Pteroscion peli* ^o^ | WJC784 |  | KX778099 | KX777878 |  |
| *Totoaba macdonaldi* ^o^ | CN717 |  | **KP722782** | **KP722963** |  |
