# Supplementary material for: Molecular exploration of hidden diversity in the Indo-West Pacific sciaenid clade
Source: PLoS One. 2017 Apr 28;12(4):e0176623. doi: 10.1371/journal.pone.0176623 (PMC5409148; doi:10.1371/journal.pone.0176623)
Supplement: S2 Table — Abbreviations of genes: COI, Cytochrome oxidase subunit I; RAG 1, Activating gene 1. Reverse primers in italics. (DOCX) [file pone.0176623.s005.docx]

**S2 Table Primers used in this study.** Abbreviations of genes: *COI*, Cytochrome oxidase subunit I; *RAG 1*, Recombination Activating Gene 1. Reverse primers in italics.

| Locus / primer | Primer sequence (5’-3’) | Source |
| --- | --- | --- |
| *COI* |  |  |
| CoxI FishF1 | TCAACCAACCACAAAGACATTGGCAC | Ward et al., 2005 |
| CoxI FishF2 | TCGACTAATCATAAAGATATCGGCAC | Ward et al., 2005 |
| Johni 76F | CCTCTGTYTRTGGGTTTACAATC | This study |
| *CoxI FishR1* | *TAGACTTCTGGGTGGCCAAAGAATCA* | Ward et al., 2005 |
| *CoxI FishR2* | *ACTTCAGGGTGACCGAAGAATCAGAA* | Ward et al., 2005 |
| *Johni 916R* | *TTRCCAGAATAATAYGCAACGA* | This study |
| *RAG1* |  |  |
| R1 2533F | CTGAGCTGCAGTCAGTACCATAAGATGT | López et al., 2004 |
| *R1 4090R* | *CTGAGTCCTTGTGAGCTTCCATRAAYTT* | López et al., 2004 |
| *R1 4061R* | *AATACTTGGAGGTGTAGAGCCAGT* | Chen et al., 2007 |

**Sources**

Chen, W.-J., Ruiz-Carus, R., Ortí, G., 2007. Relationships among four genera of mojarras (Teleostei: Perciformes: Gerreidae) from the western Atlantic and their tentative placement among percomorph fishes. J. Fish Biol. 70, 202–218.

López, J.A., Chen, W.-J., Ortí, G., 2004. Esociform phylogeny. Copeia 3, 449–464.

Ward, R.D., Zemlak, T.S., Innes, B.H., Last, P.R., Hebert, P.D.N., 2005. DNA barcoding Australia’s fish species. Phil. Trans. R. Soc. B 360, 1847–1857.
