# Supplementary material for: Molecular exploration of hidden diversity in the Indo-West Pacific sciaenid clade
Source: PLoS One. 2017 Apr 28;12(4):e0176623. doi: 10.1371/journal.pone.0176623 (PMC5409148; doi:10.1371/journal.pone.0176623)
Supplement: S4 Table — (DOCX) [file pone.0176623.s007.docx]

**S4 Table Descriptive statistics of sequences and phylogenetic performance from each locus of combined dataset.**

| Locus | ***COI*** | | | ***RAG1*** | | | **TOTAL** |
| --- | --- | --- | --- | --- | --- | --- | --- |
| Codon Position | 1st | 2nd | 3rd | 1st | 2nd | 3rd |  |
| Length^a^ (in bp) | 206 | 206 | 206 | 491 | 491 | 490 | 2090 |
| No. of variable sites (in %) | 67 (33.5%) | 19 (9.2%) | 206 (100%) | 62 (12.6%) | 37 (7.6%) | 249 (50.8%) | 640 (30.6%) |
| No. of Parsimony-informative sites | 60 | 13 | 206 | 46 | 28 | 195 | 548 |

^a^ Calculated from length of aligned DNA nucleotide sequences in base pair (bp).
